# Expression Characteristics and Functional Analysis of the *ScWRKY3* Gene from Sugarcane

**DOI:** 10.3390/ijms19124059

**Published:** 2018-12-14

**Authors:** Ling Wang, Feng Liu, Xu Zhang, Wenju Wang, Tingting Sun, Yufeng Chen, Mingjian Dai, Shengxiao Yu, Liping Xu, Yachun Su, Youxiong Que

**Affiliations:** 1 Key Laboratory of Sugarcane Biology and Genetic Breeding, Ministry of Agriculture, Fujian Agriculture and Forestry University, Fuzhou 350002, China; lingw2017@126.com (L.W.); 18359162091@163.com (F.L.); zahngxuqq7@126.com (X.Z.); wwj1470665850@163.com (W.W.); sunting3221@163.com (T.S.); CYF9410@163.com (Y.C.); 18906071567@163.com (M.D.); dbzq666666@163.com (S.Y.); xlpmail@126.com (L.X.); 2 Key Laboratory of Ministry of Education for Genetics, Breeding and Multiple Utilization of Crops, College of Crop Science, Fujian Agriculture and Forestry University, Fuzhou 350002, China

**Keywords:** sugarcane, WRKY, subcellular localization, gene expression pattern, protein-protein interaction, transient overexpression

## Abstract

The plant-specific WRKY transcriptional regulatory factors have been proven to play vital roles in plant growth, development, and responses to biotic and abiotic stresses. However, there are few studies on the *WRKY* gene family in sugarcane (*Saccharum* spp.). In the present study, the characterization of a new subgroup, IIc WRKY protein ScWRKY3, from a *Saccharum* hybrid cultivar is reported. The ScWRKY3 protein was localized in the nucleus of *Nicotiana benthamiana* leaves and showed no transcriptional activation activity and no toxic effects on the yeast strain Y2HGold. An interaction between ScWRKY3 and a reported sugarcane protein ScWRKY4, was confirmed in the nucleus. The *ScWRKY3* gene had the highest expression level in sugarcane stem pith. The transcript of *ScWRKY3* was stable in the smut-resistant *Saccharum* hybrid cultivar Yacheng05-179, while it was down-regulated in the smut-susceptible *Saccharum* hybrid cultivar ROC22 during inoculation with the smut pathogen (*Sporisorium scitamineum*) at 0–72 h. *ScWRKY3* was remarkably up-regulated by sodium chloride (NaCl), polyethylene glycol (PEG), and plant hormone abscisic acid (ABA), but it was down-regulated by salicylic acid (SA) and methyl jasmonate (MeJA). Moreover, transient overexpression of the *ScWRKY3* gene in *N. benthamiana* indicated a negative regulation during challenges with the fungal pathogen *Fusarium solani* var. *coeruleum* or the bacterial pathogen *Ralstonia solanacearum* in *N. benthamiana*. The findings of the present study should accelerate future research on the identification and functional characterization of the WRKY family in sugarcane.

## 1. Introduction

Plant growth and development are vulnerable to several external environmental challenges, such as drought, high salinity, cold, and pathogens. There are complex metabolic regulation mechanisms in plants which enhance their resistance to a wide range of stresses through physiological changes largely controlled at the molecular level [1]. The transcription factors (TFs) in plant cells interact with specific DNA sequences in target gene promoters to activate or inhibit transcription and expression of target genes, thereby regulating the expression of these genes. This modulation causes adaptation to the effects and damage from various stresses [2].

As one of the largest plant-specific families of TFs, WRKY has been proven to be widely implicated in responses to biotic and abiotic stresses [3,4]. WRKY TFs are also a vital part of the signaling pathway network of plants, regulating physiological and biochemical processes [5]. The WRKY proteins were named based on a DNA-binding WRKY domain, which contains approximately 60 amino acid residues. This domain features a WRKYGQK sequence at its N-terminal end together with a C_X45_C_X22-23_H_X_H (C_2_H_2_-type) or C_X7_C_X23_H_X_C (C_2_HC-type) zinc finger-like motif at the C-terminal [1,5,6]. Although the DNA-binding domain is highly conserved, the overall structure of the WRKY proteins is highly diverse and can be divided into three groups (I, II, and III) including five subgroups (IIa-IIe) in group II. These groupings are categorized according to the number of WRKY domains and are also based on features of the zinc finger-like motif [6,7]. Previous studies have indicated that WRKYs with similar roles usually have related functions. For example, WRKYs in groups I and III are involved in epidermal development, senescence, and abiotic stress, while WRKYs in group II are related to low phosphorus stress, disease resistance, secondary root formation, and abiotic stress, with a few exceptions observed [8].

Currently, *WRKY* genes have been identified in various plant species. There is a total of 72 *WRKY*s in the model dicot *Arabidopsis thaliana* [5]. In monocots, there are 103 *WRKY*s in *Oryza sativa* [9], 116 *WRKY*s in *Zea mays* [10], 105 *WRKY*s in *Setaria italica* [11], 68 *WRKY*s in *Sorghum bicolor* [12], and 45 *WRKY*s in *Hordeum vulgare* [13]. It has been reported that 30 *WRKY* genes in *A. thaliana* were responsive to salt stress [14]. Also, 58 *WRKY* genes in *Z. mays* and 19 *WRKY* genes in *Phaseolus vulgaris* were related to drought stress [15,16]. Qiu et al. [17] indicated that ten of 13 candidate *WRKY* genes in rice can respond to sodium chloride (NaCl), polyethylene glycol (PEG), low temperature, or high temperature stress. Wu et al. [18] showed that eight of the 15 candidate *WRKY* genes in wheat responded to low temperature, NaCl, or PEG stress. Previous studies showed that 49 *A. thaliana WRKY* genes were induced by *Pseudomonas syringae* or salicylic acid (SA) [19]. Fifteen *WRKY*s were induced by *Magnaporthe grisea*, and 12 of them were simultaneously induced by *Xanthomonas oryzae* pv. *oryzae* [9]. Several other reports also demonstrated that *WRKY*s can positively or negatively regulate the responses of plants to external biotic or abiotic stresses [20,21,22]. Moreover, numerous studies have reported that *WRKY*s are widely involved in a complicated signal transduction network, which may work together with upstream or downstream components, or may interact with other WRKY proteins during physiological processes or in response to various biotic and abiotic stimuli [1,23,24]. These reports provide the foundation for studying the tolerance mechanism of plant *WRKY* genes to environmental stress.

Sugarcane (*Saccharum* spp.) is not only the foremost sugar-producing crop, but also the one that has potential as a bioenergy resource [25]. The study on signal transduction in sugarcane growth, development, and its responses to the external environment, especially the functional analysis of TFs, is of great significance for sugarcane molecular breeding. As reported, there were 26 WRKY-like proteins discovered in a publicly available sugarcane expressed sequence tag (EST) database via an *in silico* study, and their phylogenetic relationships were determined [26]. Beyond that, only two other sugarcane group IIc WRKY proteins, Sc-WRKY (GenBank Accession No. GQ246458.1) [27] and ScWRKY4 (GenBank Accession No. MG852087.1) [28], have been isolated from *Saccharum* hybrid cultivar FN22 and *Saccharum* hybrid cultivar ROC22 respectively and characterized by molecular techniques. *Sc-WRKY* and *ScWRKY4* were both shown to be related to tolerance enhancement to PEG and NaCl stresses [27,28]. Under biotic treatment, *Sc-WRKY* may play a positive role in response to smut pathogen [27], while *ScWRKY4* may be negatively or probably not involved in this regulation [28], suggesting the functional differentiation of group IIc ScWRKYs in smut pathogen resistance. In this study, a new group IIc *WRKY* gene family member, *ScWRKY3* (GenBank Accession No. MK034706), was screened from our previous sugarcane transcriptome data [29]. The sequence characteristics of ScWRKY3 and its subcellular localization, transcriptional activation activity, and its protein-protein interaction with ScWRKY4 were analyzed. The expression profiles of *ScWRKY3* in sugarcane tissues in response to various stresses were assessed, as well as the effects that occurred in *Nicotiana benthamiana* leaves after challenging with the bacterial pathogen *Ralstonia solanacearum* and the fungal pathogen *Fusarium solani* var. *coeruleum.*

## 2. Results

### 2.1. Bioinformatics Analysis of ScWRKY3 Gene

**Figure 1:**
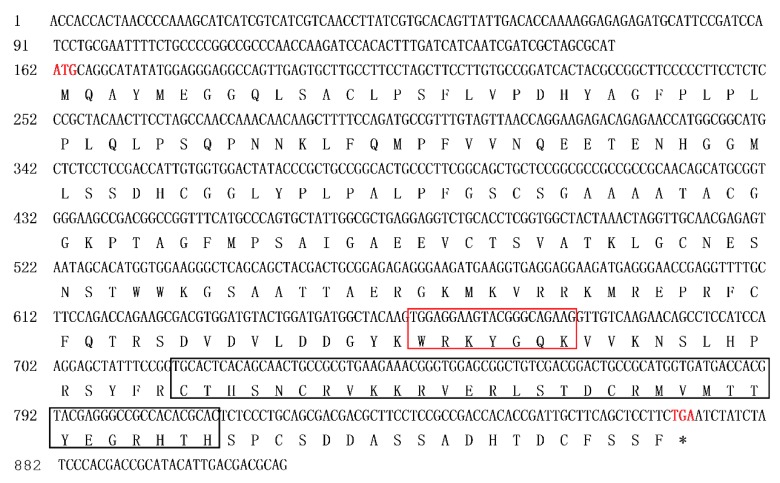
Nucleotide acid sequences and deduced amino acid sequences of the sugarcane *ScWRKY3* gene obtained by PCR amplification. The sequence of the WRKY motif (WRKYGQK) is highlighted in the red box, and that of the C_2_H_2_ domain (C_X4_C_X23_H_X_H) in the black box. The upstream sequences to start codon ATG (marked in red font) is 5′ untranslated region (UTR) and the downstream sequences to stop codon TGA (marked in red font) is 3′UTR of *ScWRKY3*. *: stop codon.

There was no nucleic acid sequence difference or amino acid sequence difference in *ScWRKY3* between smut-susceptible *Saccharum* hybrid cultivar ROC22 and smut-resistant *Saccharum* hybrid cultivar Yacheng05-179 (Figure S1). As shown in Figure 1, the *ScWRKY3* gene has a cDNA length of 910 bp containing an open reading frame (ORF) from position 162 to 872, and its encoded amino acid residues contain a conserved WRKY domain from position 166 to 223. Bioinformatics analysis revealed that the ScWRKY3 protein has a molecular weight of 25.98 kDa (Table S1). The theoretical isoelectric point (pI), grand average of hydrophobicity (GRAVY), and instability index (II) of ScWRKY3 were 8.58, -0.49, and 56.08 (Table S1), respectively, suggesting that ScWRKY3 might be an unstable basic hydrophilic protein. Secondary structure prediction showed that ScWRKY3 is mainly composed of random coil (69.07%), alpha-helix (18.22%), and extended strand (12.70%) portions (Figure S2). In addition, the ScWRKY3 protein was predicted to have no signal peptide or transmembrane domain (Figure S3). Euk-mPLoc 2.0 software [30] showed that ScWRKY3 has the highest probability of localization in the nucleus (Figure S4).

**Figure 2:**
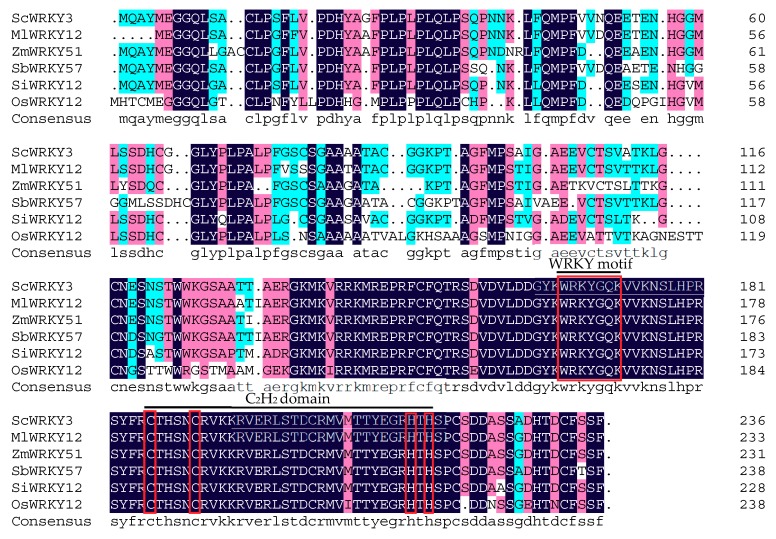
Amino acid sequence alignment of ScWRKY3 and WRKYs from other plant species by DNAMAN (version 6.0.3.99, Lynnon Biosoft) software. The amino acid sequences of *Miscanthus lutarioriparius* MlWRKY12 (AGQ46321.1), *Zea mays* ZmWRKY51 (XP_020393361.1), *Sorghum bicolor* SbWRKY57 (XP_002452824.2), *Setaria italica* SiWRKY12 (XP_004953301.1), and *Oryza sativa* OsWRKY12 (XP_015624962.1) are from GenBank. The black, pink, blue, and white colors indicate the homology level of conservation of the amino acid residues in the alignment at 100, ≥75, ≥50, and <50%, respectively. The sequences of the WRKY motif (WRKYGQK) and the C_2_H_2_ domain (C_X4_C_X23_H_X_H) are highlighted by the red rectangle.

**Figure 3:**
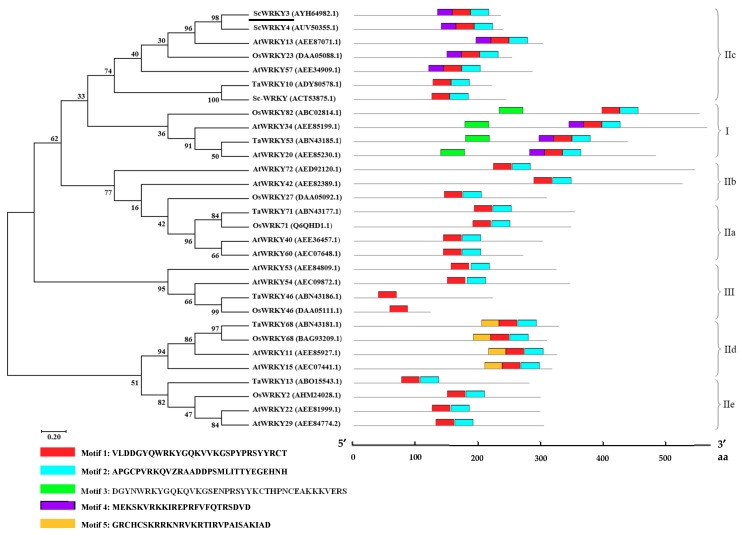
Phylogenetic tree (left) and predicted conserved motifs (right) of ScWRKY3 protein and WRKYs from various plant species. The GenBank accession number of WRKY proteins follows the protein name. Ml, *Miscanthus lutarioriparius*; Sb, *Sorghum bicolor*; Zm, *Zea mays*; Si, *Setaria italic*; Os, *Oryza sativa*; and At, *Arabidopsis thaliana.* The unrooted tree is constructed by the Maximum Likelihood with bootstrapping (1000 iterations) using MEGA7.0 software. ScWRKY3 is underlined. The conserved domains were predicted by MEME Suite 5.0.2 software. The different-colored boxes named at the bottom represent conserved motifs. Gray lines represent the nonconserved sequences, and the position of each WRKY sequence is exhibited proportionally. The motif logo is shown in Figure S6.

Amino acid sequence alignment (Figure S5) indicated that the similarity of ScWRKY3 to *S. bicolor* SbWRKY57 (XP_002452824.2), *Miscanthus lutarioriparius* MlWRKY12 (AGQ46321.1), *Z. mays* ZmWRKY51 (XP_020393361.1), *S. italica* SiWRKY12 (XP_004953301.1), *O. sativa* OsWRKY12 (XP_015624962.1) (all these accession numbers in brackets are from GenBank), sugarcane ScWRKY4 and Sc-WRKY were 93%, 93%, 87%, 87%, 66%, 53% and 24%, respectively. A conserved WRKY domain (WRKYGQK) and a conserved zinc-finger motif (C_X4_C_X23_H_X_H) at the C-terminus were found (Figure 2). The phylogenetic tree of sugarcane ScWRKY3, ScWRKY4, Sc-WRKY and WRKYs from other plant species demonstrated that WRKY proteins could be divided into three groups with no obvious distinction between monocots and dicots. ScWRKY3 was classified into group IIc, along with AtWRKY13, OsWRKY22, AtWRKY57, TaWRKY10, Sc-WRKY, and ScWRKY4 (Figure 3). MEME software prediction showed that all WRKYs except TaWRKY46 and OsWRKY46 contained motif 1 (WRKY domain) and motif 2 (zinc-finger domain). In addition, motif 3 (WRKY domain) and motif 4 (unknown domain) were detected in group I WRKYs. Some group IIc WRKYs, for example ScWRKY3, ScWRKY4, AtWRKY13, OsWRKY23 and AtWRKY57, contained motif 4. The WRKYs in group IId had their unique motif 5 (unknown domain) (Figure 3). On the whole, the phylogenetic analysis showed that most WRKYs within the same group generally had a similar structure.

### 2.2. Subcellular Localization

**Figure 4:**
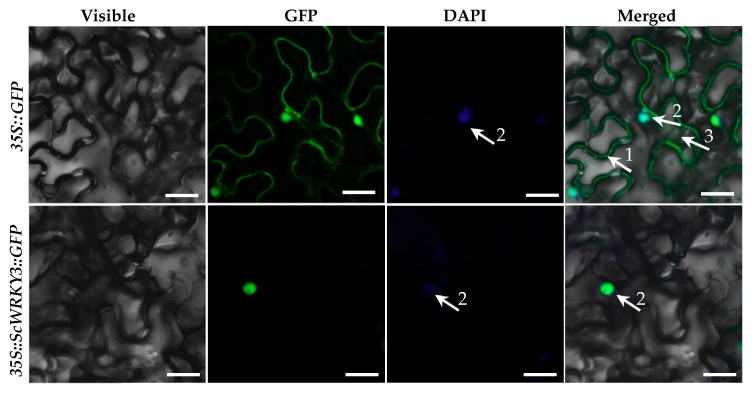
Subcellular localizations of *35S::GFP* and *35S::ScWRKY3::GFP* in *Nicotiana benthamiana* leaves. The epidermal cells of *N. benthamiana* are used for capturing images of visible light, green fluorescence, blue fluorescence, and visible light merged with green and blue fluorescence. White arrows 1, 2, and 3 indicate plasma membrane, nucleus, and cytoplasm, respectively. Scale bar = 50 μm. *35S::GFP*, the *Agrobacterium tumefaciens* strain carrying the empty vector pMDC83-*GFP*. *35S::ScWRKY3::GFP*, the *A. tumefaciens* strain carrying the recombinant vector pMDC83-*ScWRKY3-GFP*. DAPI, 4′,6-diamidino-2-phenylindole.

The recombinant vector pMDC83-*ScWRKY3-GFP* was generated to investigate the subcellular distribution of ScWRKY3. We used 4′,6-diamidino-2-phenylindole (DAPI) staining as a nuclear marker. As shown in Figure 4, the green fluorescence of the control (*35S::GFP*) in *N. benthamiana* was distributed through the whole cell, including the plasma membrane, nucleus, and cytoplasm, while the fusion protein of ScWRKY3::GFP was only found in the nucleus, which was consistent with the software prediction.

### 2.3. Transcription Activation Activity of ScWRKY3

**Figure 5:**
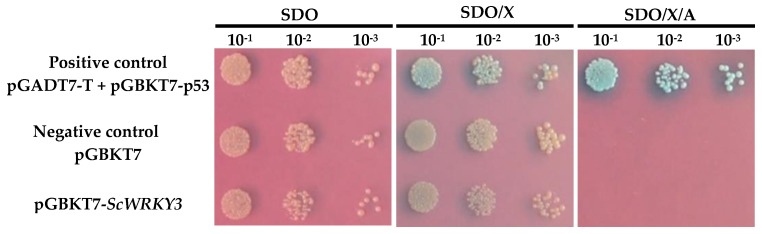
Testing of the ScWRKY3 transactivation activity assay. SDO (SD/-Trp), synthetic dropout medium without tryptophan; SDO/X (SD/-Trp/X-α-Gal), synthetic dropout medium without tryptophan, but plus 5-bromo-4-chloro-3-indoxyl-α-D-galactopyranoside; SDO/X/A (SD/-Trp/X-α-Gal/AbA), synthetic dropout medium without tryptophan, but plus 5-bromo-4-chloro-3-indoxyl-α-d-galactopyranoside and aureobasidin A.

The Y2H Gold-GAL4 yeast two-hybrid system was used to detect the transcriptional activation activity of ScWRKY3. As shown in Figure 5, yeast cells transformed with either the positive control pGADT7+pGBKT7-p53, the negative control pGBKT7-p53, or the recombinant plasmid pGBKT7-*ScWRKY3* all grew well in SDO (SD/-Trp, SD minimal medium without tryptophan) medium plates, while only the positive control turned blue in SDO/X (SD/-Trp/X-α-Gal, SDO plates with X-α-D-galactosidase) medium plates. These results indicated that all the plasmids were successfully transfected into yeast strain Y2HGold. The GAL4-BD combined with ScWRKY3 protein can successfully express tryptophan but cannot activate the *MEL1* gene in the presence of X-α-gal. After aureobasidin A (AbA) resistance screening, the yeast cells transformed with pGBKT7-*ScWRKY3* and with the negative control did not activate the two reporter genes, *AUR1-C* and *MEL1*. However, the positive control did survive, and its X-α-gal detection system showed a blue color, indicating that the ScWRKY3 protein does not possess transcriptional activation activity. This protein showed no toxicity to the yeast strain Y2HGold. This result implies that the bait protein of ScWRKY3 can be used for yeast two-hybrid screening.

### 2.4. Interaction Between ScWRKY3 and ScWRKY4

**Figure 6:**
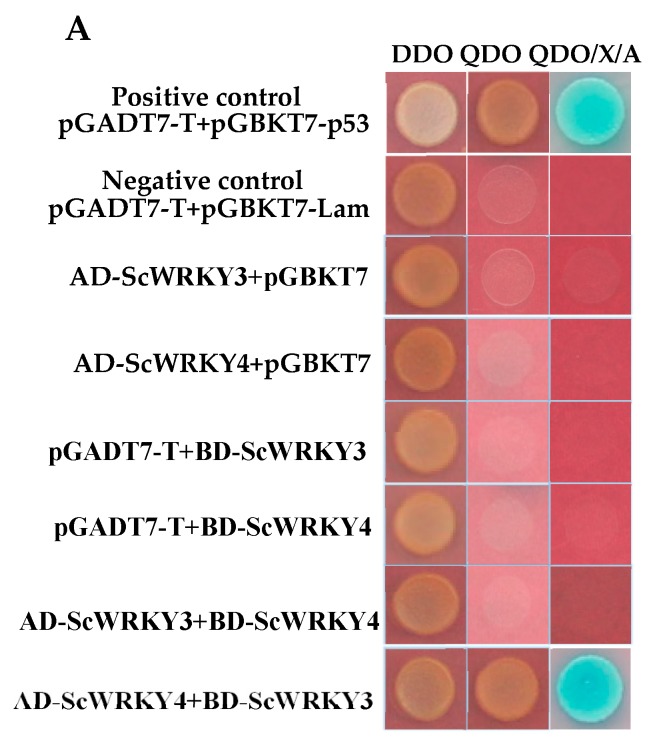

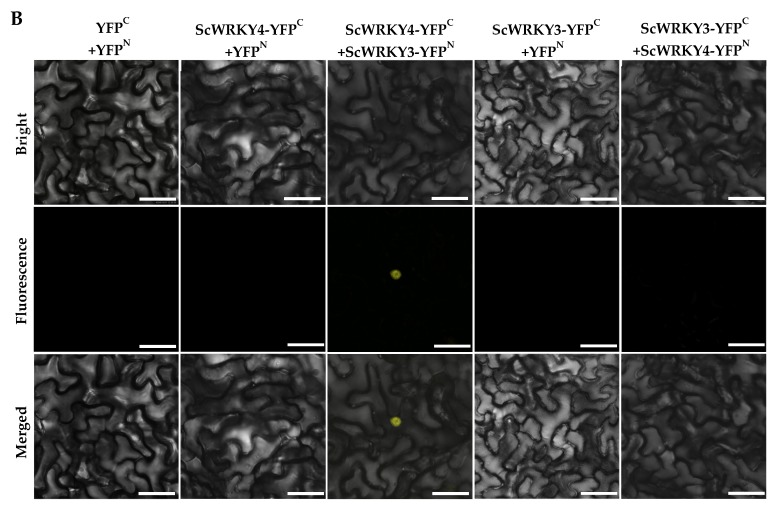
Interaction between ScWRKY3 and ScWRKY4 in yeast and in *Nicotiana benthamiana* leaves. (**A**) The interaction between ScWRKY3 and ScWRKY4 was verified using a yeast two-hybrid system. A variety of BD and AD vectors were combined and transformed into GoldY2H yeast. Left to Right: Transformations were grown and screened on DDO (SD/-Leu/-Trp, SD medium without leucine and tryptophan), QDO (SD/-Ade/-His/-Leu/-Trp, SD medium without adenine, histidine, leucine, or tryptophan), and QDO/X/A (SD/-Ade/-His/-Leu/-Trp/X-α-Gal/AbA, QDO medium with X-α-D-Galactosidase and aureobasidin medium. (**B**) The bimolecular fluorescence complementation (BiFC) assay for the location determination of the interaction between ScWRKY3 and ScWRKY4. Scale bar = 50 μm. YFP^C^, YFP^N^, ScWRKY4-YFP^C^, ScWRKY4-YFP^N^, ScWRKY3-YFP^C^, and ScWRKY3-YFP^N^ represent the plasmids pUC-SPYCE and pUC-SPYNE and the recombinant plasmids ScWRKY4-pUC-SPYCE, ScWRKY4-pUC-SPYNE, ScWRKY3-pUC-SPYCE and ScWRKY3-pUC-SPYNE, respectively.

As shown in Figure 6, all plasmid combinations grew normally on DDO (SD/-Leu/-Trp) plates. However, when transferred to QDO (SD/-Ade/-His/-Leu/-Trp) and QDO/X/A (SD/-Ade/-His/-Leu/-Trp/X-α-Gal/AbA) plates, only the AD-ScWRKY4+BD-ScWRKY3 combination and the positive control pGADT7-T+pGBKT7-p53 continued to grow and turned blue with X-α-gal detection. This indicates that ScWRKY4 may function downstream of ScWRKY3. Additionally, we have further proved the above results by bimolecular fluorescence complementation (BiFC). When ScWRKY3 was fused to pUC-SPYNE (this fusion was named ScWRKY3-YFP^N^), and ScWRKY4 was fused to pUC-SPYCE (this fusion was named ScWRKY4-YFP^C^), a fluorescent complex was formed and was visualized in the nucleus of *N. benthamiana* leaf cells. While, when ScWRKY3 was fused to pUC-SPYCE (this fusion was named ScWRKY3-YFP^C^), and ScWRKY4 was fused to pUC-SPYNE (this fusion was named ScWRKY4-YFP^N^), no fluorescent complex was formed in *N. benthamiana* leaf cells. The results were consistent with those of yeast two-hybrid system and showed that there was an interaction between ScWRKY3 and ScWRKY4, and the specific protein complex was located in the nucleus.

### 2.5. Gene Expression Patterns of ScWRKY3 in Response to Various Stress Conditions

**Figure 7:**
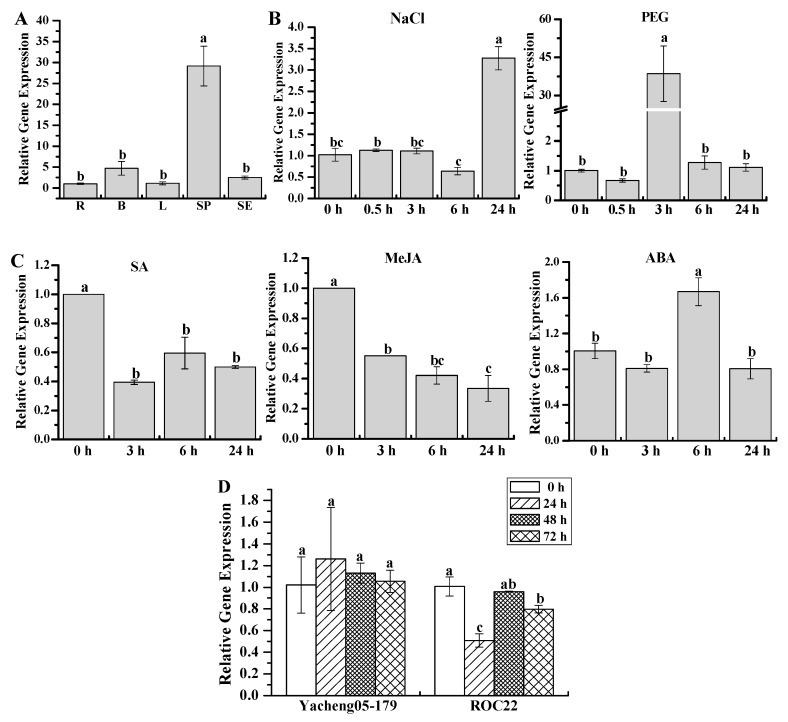
Gene expression assay of *ScWRKY3*. (**A**) Tissue-specific expression analysis of *ScWRKY3* in different 10-month-old ROC22 tissues by qRT-PCR. The tissues (root, bud, leaf, stem pith, and stem epidermis) are represented by R, B, L, SP, and SE, respectively; (**B**) Gene expression patterns of *ScWRKY3* in 4-month-old ROC22 plantlets under abiotic stress. NaCl, sodium chloride (simulating salt stress) (250 mM); PEG, polyethylene glycol (simulating drought treatment) (25.0%); (**C**) Gene expression patterns of *ScWRKY3* in 4-month-old ROC22 plantlets under plant hormone stress. SA, salicylic acid (5 mM); MeJA, methyl jasmonate (25 μM); ABA, abscisic acid (100 μM); (**D**) Gene expression patterns of the *ScWRKY3* gene after infection with smut pathogen. Yacheng05-179 is a smut-resistant *Saccharum* hybrid cultivar, and ROC22 is a smut-susceptible *Saccharum* hybrid cultivar. Data are normalized to the glyceraldehyde-3-phosphate dehydrogenase (*GAPDH*) expression level. All data points are means ± standard error (*n = 3*). Bars superscripted by different lowercase letters indicate significant differences, as determined by Duncan’s new multiple range test (*p*-value < 0.05).

The expression patterns of the *ScWRKY3* gene in sugarcane tissues and under various stresses were investigated using real-time fluorescent quantitative PCR (qRT-PCR). The results indicated that *ScWRKY3* was constitutively expressed in different sugarcane tissues, with the highest expression level in stem epidermis. It remained at lower expression levels in other tissues (root, bud, leaf, and stem pith) (Figure 7A). During the treatments with NaCl and PEG, the transcript of *ScWRKY3* in ROC22 was remarkably up-regulated by 3.28-fold at 24 h and 38.57-fold at 3 h, respectively, and remained unchanged at other time points (Figure 7B). Moreover, the expression of *ScWRKY3* in ROC22 was markedly down-regulated under both SA and methyl jasmonate (MeJA) treatments, but it was up-regulated under ABA stress with a 1.73-fold higher level than in the control (Figure 7C). These results indicated that the *ScWRKY3* gene might have positive responses to ABA, PEG, and NaCl stimuli but a negative response to SA and MeJA. After infection by the smut pathogen, the expression level of *ScWRKY3* was almost unchanged after 72 h in the smut-resistant cultivar Yacheng05-179 and during the period of 48–72 h in the smut-susceptible cultivar ROC22, while it was significantly down-regulated (0.76-fold) at 24 h in ROC22 (Figure 7D). This suggested that *ScWRKY3* may play a role in the smut pathogen response.

### 2.6. Transient Overexpression of ScWRKY3 in N. benthamiana Leaves

**Figure 8:**
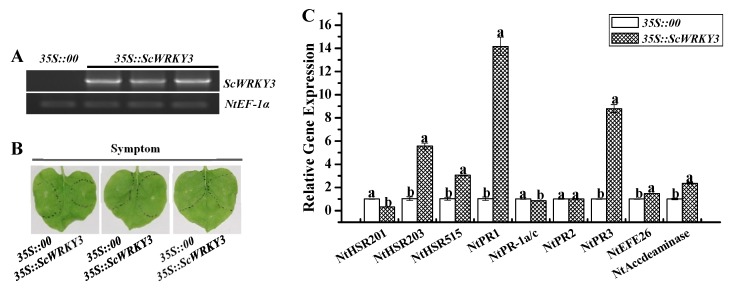

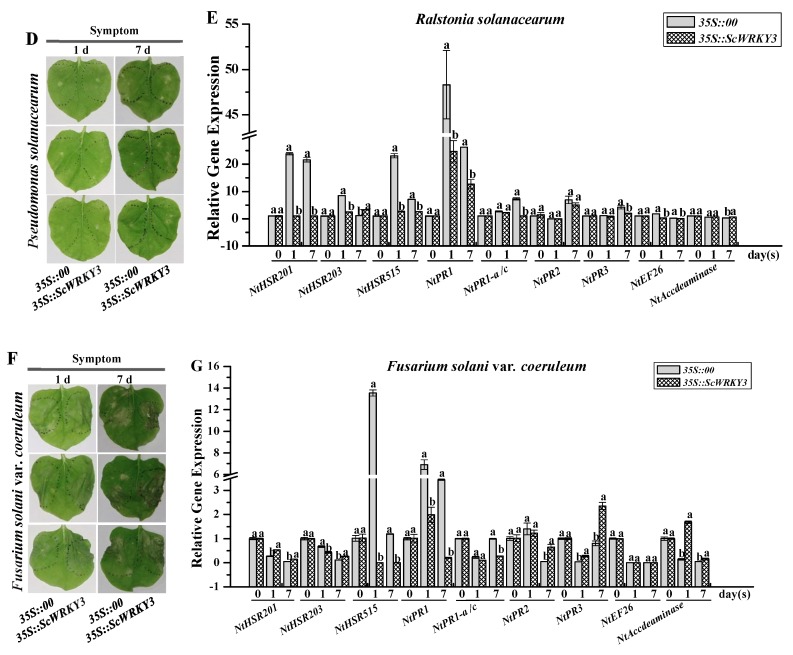
Effects of transient overexpression of *ScWRKY3* in *Nicotiana benthamiana* leaves. (**A**) Semi-quantitative PCR analysis of *ScWRKY3* in *N. benthamiana* leaves after one day of infiltration by *Agrobacterium* strain GV3101 carrying pEarleyGate 203-*ScWRKY3* (*35S::ScWRKY3*) and the empty vector pEarleyGate 203 (*35S::00*). (**B**) Phenotype of *N. benthamiana* leaves after one day of agroinfiltration. (**C**) The transcript level of nine immunity-associated marker genes in the *N. benthamiana* leaves after one day of agroinfiltration. (**D**,**F**) Disease symptoms of *N. benthamiana* post-inoculation with *Ralstonia solanacearum* and *Fusarium solani* var. *coeruleum* are observed after one day and seven days of agroinfiltration. (**E**,**G**) The transcripts of nine immunity-associated marker genes in the *N. benthamiana* leaves after inoculation with *R. solanacearum* or *F. solani* var. *coeruleum* for one day and seven days. Data are normalized to the *NtEF-1α* expression level. All data points are means ± standard error (*n = 3*). Bars superscripted by different lowercase letters indicate significant differences, as determined by Duncan’s new multiple range test (*p* value < 0.05).

The *ScWRKY3* gene was inserted into the plant overexpression vector, and was transformed into *N. benthamiana* leaves by the *Agrobacterium tumefaciens*-mediated method to analyze whether the target gene could induce a plant immune response. The transcripts of *ScWRKY3* in *N. benthamiana* leaves were detected using a semi-quantitative PCR technique (Figure 8A). The phenotypic observation after injection for one day was shown in Figure 8B, and no significant difference in superficial characteristics was demonstrated between the experimental group and the control group. However, qRT-PCR results demonstrated that six immunity-associated marker genes, including the hypersensitive response (HR) marker genes, *NtHSR203* and *NtHSR515*, the SA pathway related gene *NtPR1*, the JA pathway associated gene *NtPR3,* and two ethylene synthesis-dependent genes, *NtEFE26* and *NtAccdeaminase*, were all up-regulated with a higher fold change range from 1.47 to 14.16 than the control (Figure 8C). These results suggest that transiently overexpressed *ScWRKY3* may take part in the immune response in *N. benthamiana* leaves.

To detect the effect of *ScWRKY3* in response to pathogen, the *N. benthamiana* leaves, were transformed with the control in the left half blade and with *35S::ScWRKY3* in the right half blade for one day. Then they were inoculated by the bacterial pathogen *R. solanacearum*. As shown in Figure 8D, there was a slight symptomatic difference between the control half-leaves and the *35S::ScWRKY3* half-leaves when injected with *R. solanacearum* for one day and seven days. Moreover, qRT-PCR results revealed that the HR marker genes *NtHSR201* and *NtHSR515* and the SA-related gene *NtNPR1* all showed significantly lower expression in *35S::ScWRKY3*-overexpressing *N. benthamiana* leaves after one day and seven days of *R. solanacearum* inoculation when compared to the control. No remarkable transcript difference or down-regulation of the SA-related gene *NtPR-1a/c* or the JA-associated genes *NtPR2* and *NtPR3* was observed in *35S::ScWRKY3* leaves when compared with controls. Compared to the control, the transcript abundance of *NtHSR203* in *35S::ScWRKY3*-overexpressing leaves was decreased at one day but increased at seven days post-agroinfiltration.

When the leaves of *ScWRKY3-*transiently-overexpressing *N. benthamiana* were inoculated by the fungal pathogen *F. solani* var. *coeruleum* for one day and seven days, a heavier wilting disease symptom was observed in the *N. benthamiana* leaves containing *35S::ScWRKY3* than in the control (Figure 8F). Additionally, in comparison with the control, *NtHSR201*, *NtPR3,* and *NtAccdeaminase* showed significantly higher expression, while *NtHSR515* and *NtNPR1* presented obviously lower expression in *35S::ScWRKY3*-overexpressing leaves at one day or seven days after *F. solani* var. *coeruleum* infection. No statistically significant expression difference in *NtPR-1a/c*, *NtPR2,* or *NtEFE26* was found at one day, while *NtPR-1a/c* was down-regulated, *NtPR2* was up-regulated, and *NtEFE26* was unchanged in *35S::ScWRKY3* leaves after seven days with *F. solani* var. *coeruleum* inoculation. The expression level of *NtHSR203* in *35S::ScWRKY3* was lower at one day but higher at seven days after inoculation than in the control (Figure 8G).

These results demonstrated that in comparison with the transiently overexpressing pEarleyGate 203 vector, *ScWRKY3* transient overexpression in *N. benthamiana* leaves significantly decreased the transcript abundance of *NtHSR515, NtPR1,* and *NtPR-1a/c* after *R. solanacearum* or *F. solani* var. *coeruleum* infection. It was anticipated that *ScWRKY3* can negatively regulate the HR marker genes or SA signaling pathway-mediated genes to reduce the tolerance of *N. benthamiana* to pathogens.

## 3. Discussion

As one of the largest groups of TFs, the WRKY proteins have been found in a wide range of plant species since the initial *WRKY* cDNA was isolated from sweet potato [31]. Although sugarcane is an important bioenergy and cash crop [25], there are only three reports about WRKYs in sugarcane [26,27,28]. In this study, a novel sugarcane *ScWRKY3* gene was isolated and identified. As reported, there is a functional similarity of WRKYs in the same or phylogenetically closely related group [2,3]. Phylogenetic tree analysis indicated that ScWRKY3 is a member of the group IIc WRKY proteins, along with Sc-WRKY [27] and ScWRKY4 [28] (Figure 3). This is helpful for further functional comparative studies on the same WRKY family members in sugarcane. The structure of TFs is usually composed of four functional domains, namely, the DNA binding domain, the transcriptional activation or repression domain, the oligomerization sites, and the nuclear localization signals [32]. These four components are the core regions that perform the functions of TFs or interact with the *cis*-acting elements in the promoter regions of various stress related genes [33]. In our study, ScWRKY3 protein had one WRKY domain, which is a DNA binding domain containing 60 amino acids. WRKY domain was mainly composed of motif 1 and motif 2 (Figure 3) and can bind specifically to the DNA sequence motif (T)(T)TGAC(C/T) which is known as the W-box and existed in many promoters of plant defense-related genes [6]. As showed by Wei et al. [10], subgroup IId WRKYs possess two basic amino acid sequences, including a RCHCSK[RK][RK]K[LN]R motif, which may function as a nuclear localization signal, and a KRxIxVPAISxKxAD motif. Similarly, the motif 5 (Figure 3) also contains these amino acid sequences. While further work is required to clarify the function of the other unknown motifs, such as the predicted motif 4 in Figure 3. Previous studies showed that the functions of the two WRKY domains in the group I WRKYs are different. The WRKY domain at the C-terminal can bind to their target DNA, while another WRKY domain at the N-terminal may be as the site where proteins interact with each other [8,12,15].

Subcellular localization analysis is valuable for determining the functions of proteins. The present study showed that the fusion protein of ScWRKY3::GFP was detected in the nucleus of *N. benthamiana* leaf cells (Figure 4), which was consistent with the software prediction results and previous studies on other plant WRKYs [28,34,35,36]. This indicated that ScWRKY3 may play a role as a nuclear-localized protein to regulate cellular processes.

Transcriptional activity analysis is important for the functional analysis of TFs [37]. Since the GAL4 yeast two-hybrid system was first discovered [38], this method has been increasingly used to study the interactions between WRKY proteins [39]. In this study, the full-length ScWRKY3 cDNA showed no auto-activation (Figure 5), so it could be used as the bait to screen interacting proteins in a yeast two-hybrid system. Post-translational modifications or interactions with cofactors are needed for ScWRKY3 protein to fulfill its function. Screening and identifying WRKY interacting proteins is important to reveal the role of WRKY in plant signal transduction [40,41]. It has been reported that WRKYs have the activities of self-regulation and mutual regulation, and they can form functional homo- or heterodimers among some WRKY proteins or interact with other functional proteins to play roles [4,5]. WRKY6 and WRKY22, which both belong to group II WRKYs in *A. thaliana*, interact with MPKl0 and MPK3/MPK6, respectively [42,43]. Previous studies also proved that AtWRKY30, AtWRKY53, AtWRKY54, and AtWRKY70, which all belong to group III of the WRKY proteins, have interactive effects in yeast [39]. Similarly, yeast two-hybrid and BiFC results showed that a fluorescent complex from ScWRKY3 and ScWRKY4 was formed (Figure 6B). This was visualized in the nucleus in *N. benthamiana* leaf cells, which indicated that there may be an interacting relationship between ScWRKY3 and ScWRKY4. In *Arabidopsis*, previous study indicated that WRKYs in the group IIb or group III could interact with themselves and with group IIa WRKYs, while group IId WRKYs could only interact with group IIa WRKY members [41]. Groups IIc ZmWRKY25 and ZmWRKY47 had interactions and may be involved in the response to drought stress by interacting with other WRKYs [15]. Besides, ZmWRKY39 was down-regulated under light drought stress and its phylogenetically closely related protein ZmWRKY106 showed a positive response to this stress, while they had up-regulated co-expression interaction under drought stress [15]. However, the nature of the interaction as well as the biological implications of this process between ScWRKY3 and ScWRKY4 requires further study.

*WRKY* genes are expressed differentially in different tissues of plants, which demonstrates that *WRKY* genes can be expressed in different physiological conditions and in different types of cells, and may regulate a series of life activities including growth, development, and morphological composition [35,40,44]. Twenty-eight *CiWRKY*s were detected in the roots, stems, and leaves of wild *Caragana intermedia,* and different *CiWRKY*s showed differential expression in various tissues. For example, *CiWRKY69–1* had the highest expression level in roots, while *CiWRKY40–1* and *CiWRKY30* were mainly expressed in leaves [35]. Among the 37 *A. thaliana WRKY* genes reported by Bakshi et al. [45], 12 were specifically expressed in the mature zone of root cells, suggesting that these *WRKY* genes may be involved in the regulation of root cell maturation in *A. thaliana*. *TaWRKY44*, a *WRKY* gene of *Triticum aestivum*, was differentially expressed in all organs examined, including root, stem, leaf, pistil, and stamen, with the highest expression level in the leaves and the lowest expression level in the pistils [40]. It is known that *ScWRKY4* is constitutively expressed in the root, bud, leaf, stem pith, and stem epidermis of sugarcane, with the highest expression level in the stem epidermis [28]. This was similar to the findings on tissue-specific expression of *ScWRKY3* in the present study. Can the coexpression of *ScWRKY3* and *ScWRKY4* in the same tissue be tied together with their BiFC interaction results (Figure 6B)? Can they co-localize in the same organelle in the same tissue? These remain to be validated by future research, for example, only if we obtain the promoters specific to *ScWRKY3* and *ScWRKY4* respectively, can we check that if they co-localize in the same organelle in the same tissue. WRKY TFs are critical for signal transduction, plant growth and stress responses [44]. Salt and drought, the two representative abiotic stresses, adversely affect the growth and development of plants, but the response can be resisted by activating the ABA signal transduction pathway to induce the expression of a series of stress-responsive genes [46]. *AtWRKY1* played a negative role in ABA-mediated drought resistance, and the *AtWRKY1* knockout mutant could enhance the drought tolerance of *A. thaliana* [47]. In the *OsWRKY11* knockout mutant, drought responsive genes were induced to enhance the drought tolerance of rice [36]. The present study showed that the expression of *ScWRKY3* was increased by PEG, NaCl, and exogenous ABA. Previous studies also determined that *Sc-WRKY* and *ScWRKY4* showed up-regulated expression levels under PEG and NaCl treatments [27,28]. Furthermore, under ABA stress, the transcript of *ScWRKY4* was remarkably up-regulated by 1.59-, 2.87-, and 1.26-fold at 0.5 h, 6 h, and 24 h higher than the control, respectively [28]. These results suggest that *ScWRKY3* and *ScWRKY4* may participate in sugarcane resistance to drought and salt stresses which may be mediated through ABA signaling. Similar to the expression characteristics seen in group II *WRKYs* of other plants, the expression level of *AtWRKY57* was remarkably up-regulated in drought conditions. This occurred through the direct activation of the expression of an important functional gene (*AtNCED3*) in the ABA synthesis pathway, which enhanced the tolerance of *A. thaliana* to drought stress [48].

Plants have evolved at least two sets of biochemical defenses to protect themselves against external challenges [49]. One defense response, caused by infection of pathogenic bacteria, initiates a localized hypersensitive reaction to confine the injured site and to prevent further infection of pathogens. This is known as systemic acquired resistance (SAR) [50]. The other response is mainly activating the expression of defense genes through various signal molecules to exhibit resistance in plants. The signal transduction pathways related to this defense response may be mediated by SA, JA, or ABA [51,52]. Previous studies indicated that the *WRKY* genes of group IIc are related to plant immunity. For instance, *AtWRKY28* and *AtWRKY75* can be induced by infection with *Sclerotinia sclerotiorum* in connection with SA- and JA/ET-mediated defense signaling pathways [53]. Overexpression of rice *WRKY89* increased the expression level of SA and enhanced the resistance to rice blast fungus [54]. *AtWRKY57* is negatively associated with resistance to *Botrytis cinerea* in *A. thaliana* by regulating the expression of JA pathway-related genes [49]. *OsWRKY13* enhances the resistance of rice to *X. oryzae* pv. *oryzae* and *M. grisea* [55,56]. In the present study, *ScWRKY3* was down-regulated by smut pathogen in the smut-susceptible cultivar ROC22 at 24 h, while it was almost unchanged in the smut-resistant cultivar Yacheng05-179. Conversely, Wang et al. [28] showed that the expression of *ScWRKY4* was quite stable in ROC22 but was down-regulated in Yacheng05-179 under the stress of smut pathogen. Liu et al. [27] found that the expression of *Sc-WRKY* was remarkably up-regulated at 24 and 60 h after infection by smut pathogen treatment in sugarcane FN22. Moreover, *ScWRKY3* was down-regulated under SA and MeJA treatments, which was opposite to the expression patterns of *ScWRKY4* and *Sc-WRKY* [27,28]. The results revealed that *ScWRKY3* might be a negative regulatory gene in the sugarcane response to smut pathogen. This can be further proved by antimicrobial test in more stable overexpression and knockout plants. In *Arabidopsis*, *AtWRKY25* gene was proved to play a negative regulatory role in the SA-mediated defense response to *Pseudomonas syringae* [57] but a positive regulatory role in response to NaCl stress [58]. Yokotani et al. [59] demonstrated that overexpression of *OsWRKY76* in rice plants suppressed the induction of defense related genes after inoculation with blast fungus (*Magnaporthe oryzae*) but up-regulated the expressions of abiotic stress-associated genes using microarray analysis. It is therefore possible for *WRKY* genes to play opposite roles in biotic resistance and abiotic tolerance. In the future, genetic transformations can be done to further our understanding on the responses of *ScWRKY* genes to biotic and abiotic stresses. In addition, whether there are sequence differences in the promoter regions of the *ScWRKY* gene*s* from different sugarcane cultivars which may cause differences in gene expression patterns for the biotic or abiotic stress need further investigation.

As Liu et al. [60] proved, overexpression of the *Gossypium hirsutum GhWRKY25* in *N. benthamiana* is involved in the regulation of expression of multiple defense-associated marker genes, including the SA-, ET-, and JA-mediated genes, to decrease the resistance to the fungal pathogen *B. cinerea*. *GhWRKY40* has been revealed to be inducible by stress from the bacterial pathogen *R. solanacearum*, and *GhWRKY40* expression was up-regulated by SA, MeJA, and ET [61]. When *GhWRKY40* was transiently overexpressed in *N. benthamiana* leaves, most of the resistance-associated genes, including the SA-, ET-, JA-, and HR-responsive genes, were down-regulated after infection with *R. solanacearum*, which indicated that overexpression of *GhWRKY40* reduces the tolerance to *R. solanacearum* [61]. *A. thaliana* WRKY27 negatively regulated resistance genes during infection with the pathogen *R. solanacearum*, and the symptom development in *R. solanacearum* appeared earlier than in the mutant wrky27-1 plants, which lacks the function of WRKY27 [62]. In this study, most of the immunity-associated marker genes, including the HR-, SA-, JA-, and ET-related genes, were up-regulated when *ScWRKY3* was transiently overexpressed in *N. benthamiana,* suggesting that *ScWRKY3* may play a role in the plant immune response. After infection with the bacterial pathogen *R. solanacearum*, most of the detected immunity-associated marker genes, including the HR marker genes *NtHSR201* and *NtHSR515*, the SA-related genes *NtPR-1a/c* and *NtNPR1*, and the JA-associated gene *NtPR3*, were lower in the *35S::ScWRKY3* overexpressing leaves than in the control, revealing that *ScWRKY3* may play a negative role in the response to the bacterial pathogen *R. solanacearum*. After *F. solani* var. *coeruleum* infection, the wilting disease symptoms were greater in *35S::ScWRKY3* leaves than in the control. The qRT-PCR results showed that the transcript abundance of JA- and ET-related genes was remarkably higher, but the SA-related genes were evidently reduced in *35S::ScWRKY3* leaves compared to their levels in the control. These results indicated that there was a crosstalk between JA-/ET-related genes and SA-related genes in the response to the fungal pathogen *F. solani* var. *coeruleum* when *ScWRKY3* was overexpressed in *N. benthamiana*.

## 4. Materials and Methods

### 4.1. Plant Materials and Treatments

In China, ROC22 has been the main sugarcane cultivar grown for the past 20 years and encompasses approximately 60% of the total sugarcane cultivated area. ROC22 is a *Saccharum* hybrid cultivar which is susceptible to smut disease and results in a poor ratoon performance. Yacheng05-179, an intergeneric hybrid (BC2) with smut resistant properties, is generated from *S. officinarum* × *S. arundinaceum*. In this study, ROC22 and Yacheng05-179 were used as plant materials and collected from the Key Laboratory of Sugarcane Biology and Genetic Breeding, Ministry of Agriculture, Fuzhou, China.

To analyze the tissue-specific expression level of the target gene, nine healthy and uniform 10-month-old ROC22 plants were randomly selected from one field. The white root, bud, +1 leaf, stem pith, and stem epidermis were immediately frozen in liquid nitrogen and kept at -80 °C until extraction of total RNA. Each sample contained three biological replicates.

For biotic treatment, the robust and healthy stems of 10-month-old ROC22 and Yacheng05-179 were harvested and soaked in water for germination at 32 °C. Then the two-bud setts of both sugarcane cultivars were inoculated with 0.5 µL suspensions of 5 × 10^6^ smut spores/mL (plus 0.01% (*v/v*) Tween-20), while the control was inoculated with aseptic water in 0.01% (*v/v*) Tween-20 [63]. The treated samples were cultured at 28 ± 1 °C in a photoperiod of 16-h light and 8-h darkness. Three biological replicates were set, and five buds were randomly chosen at 0 h, 24 h, 48 h, and 72 h for each biological replicate, respectively.

For abiotic and hormone stimuli, healthy and uniform approximately 4-month-old ROC22 plantlets were transferred to water for one week and then treated with six different exogenous stresses. Two groups were separately cultured in aqueous solutions of 250 mM NaCl and 25% PEG 8000, and the leaves were sampled at 0, 0.5, 3, 6, and 24 h, respectively [63,64,65]. The other three groups were sprayed with 100 μM ABA, 5 mM SA in 0.01% (*v/v*) Tween-20 and 25 μM MeJA for 0, 3, 6, and 24 h, respectively [63,64,65]. Each treatment was prepared with three biological replicates that contained three plants. All collected samples were immediately frozen in liquid nitrogen and kept at −80 °C until use.

### 4.2. RNA Extraction and First-strand cDNA Synthesis

The total RNAs of all the samples were extracted with TRIzol^®^ reagent (Invitrogen, Shanghai, China). The RNA quality was determined by 1.0% agarose gel electrophoresis and measured at wavelengths of 260 and 280 nm using a spectrophotometer (NanoVueplus, GE, USA). The residual DNA was removed by DNase I (Promega, Madison, WI, USA). The RevertAid First Strand cDNA Synthesis Kit (Fermentas, Shanghai, China) was used to synthesize the first-strand cDNA from ROC22 and Yacheng05-179 leaves which was treated as templates for cloning the target gene. Prime-Script™ RT Reagent Kit (Perfect Real Time) (TaKaRa Biotechnology, Dalian, China) was used to synthesize the first-strand cDNA of the other samples for expression profile analysis.

### 4.3. Cloning, Sequencing, and Bioinformatic Analysis of the ScWRKY3 Gene

A gene which codes for a predicted *WRKY* transcriptional regulator named *ScWRKY3* was screened from our previous transcriptome data of sugarcane infected by smut fungus [29]. The specific amplification primers (Table S2) were designed using National Center of Biotechnology Information (NCBI) online software (https://www.ncbi.nlm.nih.gov/tools/primer-blast/). The reverse transcription-polymerase chain reaction (RT-PCR) system contained 1.0 µL cDNA template, 1.0 μL each of the forward and reverse primers (10 μM), 2.5 μL 10× ExTaq buffer (Mg^2+^ plus), 2.0 μL dNTPs (2.5 mM), and 0.125 μL ExTaq enzyme (5.0 U/μL) (TaKaRa Biotechnology, Dalian, China), and 17.375 μL ddH_2_O. The RT-PCR reaction conditions were as follows: 94 °C for 4 min; 35 cycles of 94 °C for 30 s, 58 °C for 30 s, and 72 °C for 1 min 30 s; and 72 °C for 10 min. The amplified fragment, which had been gel-purifed using a Gel Extraction Kit (Tiangen, Beijing, China), was linked to the pMD19-T vector (TaKaRa Biotechnology, Dalian, China) and transformed into *Escherichia coli* strain DH5*α* cells. The positive clones were selected for sequencing (Biosune, Fuzhou, China).

The sequence of the *ScWRKY3* gene was analyzed using the ORF Finder (https://www.ncbi.nlm.nih.gov/orffinder/) and a conserved domains program (http://www.ncbi.nlm.nih.gov/Structure/cdd/wrpsb.cgi) [66]. ProtParam (https://web.expasy.org/protparam/) [67] and NPS@ srever (https://npsa-prabi.ibcp.fr/cgi-bin/npsa_automat.pl?page=/NPSA/npsa_hnn.html) [68] were used for analyzing the primary structure and secondary structure of the ScWRKY3 protein, respectively. The online programs SignalP 4.1 Server (http://www.cbs.dtu.dk/services/SignalP/) [69,70], TMHMM Server v. 2.0 (http://www.cbs.dtu.dk/services/TMHMM/) [71], and Euk-mPLoc 2.0 Server (http://www.csbio.sjtu.edu.cn/bioinf/euk-multi-2/) [30] were used to predict the signal peptide, the transmembrane domain, and the subcellular localization of the target protein, respectively. The BLASTp program (https://blast.ncbi.nlm.nih.gov/Blast.cgi?PROGRAM=blastp&PAGE_TYPE= BlastSearch&LINK_LOC=blasthome) in NCBI was used to find the homologous amino acid sequences from other plants. The multiple alignment was performed using DNAMAN 6.0.3.99 software. Then the MEGA 7.0 software [72] with the Maximum Likelihood (ML) (1000 BootStrap) method was used to construct the unrooted phylogenetic tree of ScWRKY3 with sugarcane Sc-WRKY, ScWRKY4, and WRKY proteins from *Arabidopsis thaliana* [14] and other plants [13,73]. The online software MEME Suite 5.0.2 (http://meme.sdsc.edu/meme/intro.html) [73] was used to build the logo representations of the conservative domain and the rest of the alignment.

### 4.4. Subcellular Localization

The complete coding region of *ScWRKY3* without a stop codon was amplified using the primers *ScWRKY3*-Gate-F and *ScWRKY3*-Gate-R (Table S2), which were designed based on the sequences of *ScWRKY3* and the Gateway^®^ donor vector of pDONR221. The gel-purified product was linked into pDONR221 using the Gateway BP Clonase^TM^ II enzyme mix (Invitrogen, Carlsbad, CA, USA) and transformed into DH5*α* cells and sequenced (Biosune, Fuzhou, China). The Gateway LR Clonase^TM^ II enzyme mix (Invitrogen) was used to ligate pDONR221-*ScWRKY3* into the subcellular localization vector pMDC83-*GFP* [74]. GV3101 cells, carrying the recombinant vector pMDC83-*ScWRKY3-GFP* or the pMDC83-*GFP* vector were inoculated into LB liquid medium supplemented with 35 μg/mL rifampicin and 50 μg/mL kanamycin, and shaken overnight in an incubator at 200 rpm and 28 °C. Subsequently, Murashige and Skoog (MS) liquid medium was used to dilute the cell density of the *Agrobacterium* solutions to an OD_600_ of 0.8. This was supplemented with 200 μM acetosyringone and cultured in the dark for 30 minutes. Then the *Agrobacterium* solutions were injected into the leaves of eight-leaf stage *N. benthamiana* using a 1.0 mL sterilized syringe [75,76]. After two days of infiltration, the treated leaves were collected and stained with 1.0 µg/mL DAPI solution in dark conditions for one h. The subcellular localization result was observed using a Leica Microsystems microscope (model Leica TCS SP8, Mannheim, Germany) with a 10 × lens, a chroma GFP filter set for EGFP (excitation at 488 nm), and a DAPI filter set for chromatin (excitation at 458 nm) [60].

### 4.5. Analysis of Transcriptional Activation of ScWRKY3 in Yeast Cells

To analyze the transcriptional activation of ScWRKY3, the Y2HGold-GAL4 yeast two hybrid system (containing four reporter genes, including *AUR1-C*, *HIS3*, *ADE2,* and *MEL1*) was used following the manufacturer’s instructions for the Matchmaker Gold yeast two-hybrid system [49]. The *ScWRKY3* gene was PCR-amplified from pMD19-T-*ScWRKY3* using primers *ScWRKY3*-BD-F and *ScWRKY3*-BD-R (Table S2). The gel-purified product was double-digested with *Nde* I and *Bam*H I enzymes, as was the plasmid pGBKT7. Then the recombinant plasmid pGBKT7-*ScWRKY3* was constructed using T4 DNA ligase (5 U/µL) (Thermo Fisher, Shanghai, China). The pGBKT7 vector, containing the nutritional screening marker gene *TRP1*, was used as a negative control. Plasmids of pGBKT7-53+pGADT7-T have been proven to bind the 53 protein and the T protein in yeast cells. The hybrid vector can activate the reporter gene *AUR1-C* on a plate that contains the AbA antibiotics, so it was used as the positive control. The empty vector plasmid pGBKT7 and plasmids pGBKT7-53+pGADT7-T and pGBKT7-*ScWRKY3* were transformed into yeast strain Y2HGold following the manufacturer’s protocol for Y2HGold Chemically Competent Cells (TaKaRa Biotechnology, Dalian, China). The positive colonies were screened from selective medium plates for transferring onto the SDO (SD/-Trp, SD minimal medium without tryptophan), the SDO/X (SD/-Trp/X-α-Gal, SDO plates with X-α-D-Galactosidase), and the SDO/X/A (SD/-Trp/X-α-Gal/AbA, SDO/X plates with aureobasidin A) plates, respectively. Then the transcriptional activation activities were calculated by observing and imaging the growth conditions of the yeast cells after incubating for 2–3 days in a 29 °C incubator.

### 4.6. Analysis of Interaction Between ScWRKY3 and ScWRKY4

Transcriptional activation analysis in this study and a previous study [28] showed that ScWRKY3 and ScWRKY4 (GenBank Accession No. AUV50355.1) did not possess transcriptional activation activity, and the bait protein has no toxic effect on the yeast strain Y2HGold. Hence, the interacting relationship between ScWRKY3 and ScWRKY4 was identified by a yeast two-hybrid system and BiFC analysis. In the yeast two-hybrid system, AD-ScWRKY3 or AD-ScWRKY4 was used as a prey vector, and BD-ScWRKY4 or BD-ScWRKY3 was used as a bait vector, respectively. A double-enzyme digestion method was used for bait vector and prey vector construction. The specific primers with corresponding restriction enzyme sites are shown in Table S2. pGADT7-T was used as prey control, and pGBKT7-p53 or pGBKT7-Lam was used as the positive or negative bait control, respectively. These combination constructs, including the positive control pGADT7-T + pGBKT7-p53, the negative control pGADT7-T + pGBKT7-Lam, AD-ScWRKY4 + pGBKT7, AD-ScWRKY3 + pGBKT7, pGADT7-T + BD-ScWRKY3, pGADT7-T + BD-ScWRKY4, AD-ScWRKY3 + BD-ScWRKY4, or AD-ScWRKY4 + BD-ScWRKY3, were co-transformed into yeast strain Y2HGold following the manufacturer’s protocol for Y2HGold Chemically Competent Cells (TaKaRa Biotechnology, Dalian, China). Subsequently, the transformed yeast cells were selected using yeast selective medium DDO (SD/-Leu/-Trp) to detect whether all the plasmids were successfully transfected into the yeast strain Y2HGold. Then the interaction between ScWRKY3 and ScWRKY4 was detected using QDO (SD/-Ade/-His/-Leu/-Trp) and QDO/X/A (SD/-Ade/-His/-Leu/-Trp/X-α-Gal/AbA) medium [64]. For BiFC vector construction, we used the Gateway method [77]. The coding sequences of *ScWRKY3* and *ScWRKY4* were amplified and linked into the non-fluorescent fragment in the pUC-SPYNE or pUC-SPYCE vector through LR-recombination using Gateway primers (Table S2). The two cooperating plasmids were transformed into the *N. benthamiana* leaves using the *Agrobacterium*-mediated method [78,79]. After five days of infiltration, the presence of fluorescence from yellow fluorescent protein (YFP) was observed using Leica Microsystems (model Leica TCS SP8, Mannheim, Germany) with a 10 × lens and a YFP filter (excitation at 561 nm).

### 4.7. Expression Patterns of ScWRKY3 in Sugarcane Tissues under Various Stresses

For the expression pattern analysis of *ScWRKY3* in sugarcane tissues (root, bud, leaf, stem pith, and stem epidermis) and in response to various stresses (NaCl, PEG, SA, MeJA, and ABA), the qRT-PCR primers ScWRKY3-QF/R (Table S2) were designed using the Beacon Designer V8.14 software. Glyceraldehyde-3-phosphate dehydrogenase (*GAPDH*) (GenBank Accession Number: CA254672) was used as the reference gene (Table S2). An ABI 7500 Real-Time PCR System (Applied Biosystems, Foster City, CA, USA) and a SYBR Green PCR Master Mix Kit (Roche, Shanghai, China) were used for qRT-PCR analysis. The qRT-PCR reaction system was subjected to 50 °C for 2 min, 95 °C for 10 min, 95 °C for 15 s, and 59 °C for 1 min, for 40 cycles. A melting curve analysis was performed at 95 °C for 15 s, 60 °C for 1 min, 95 °C for 15 s, and 60 °C for 30 s. Each sample was set up for triplicate technical replicates, and sterile water was used as the negative control template. The 2^−∆∆*C*^_T_ method [80], DPS 9.50 software, and Origin 8 software were adopted to calculate the relative expression of the target gene (Tables S3–S6), to analyze the significance level of the experimental data, and to structure the histogram, respectively. To reduce the effects of mechanical injury on the expression of the target gene in the inoculation test with the smut pathogen, the relative expression level of the *ScWRKY3* gene was determined by subtracting the expression of the sterile water at the corresponding time according to Su et al. [81].

### 4.8. Transient Expression of ScWRKY3 in N. benthamiana

The Gateway LR Clonase^TM^ II enzyme mix (Invitrogen) was used to ligate pDONR221-*ScWRKY3*, as mentioned above, into the overexpression vector of pEarleyGate 203 [82]. The plasmid of pEarleyGate 203-*ScWRKY3* was transformed from the Gateway LR reaction into *A. tumefaciens* strain GV3101, while the pEarleyGate 203 vector which was transformed into GV3101 alone was used as a control. GV3101 cells were shaken overnight in LB liquid medium supplemented with 35 μg/mL rifampicin and 50 μg/mL kanamycin at 200 rpm and 28 °C. The *Agrobacterium* solutions were collected and resuspended to OD_600_ = 0.8 using the MS liquid medium and supplemented with 200 μM acetosyringone. Then the *Agrobacterium* suspensions were injected into the lower epidermis of the eight-leaf stage of *N. benthamiana* leaves using a 1.0 mL sterilized syringe and cultured at 28 °C with a photoperiod of 16-h light and 8-h darkness [76]. Each group of the injected leaves was collected for RNA extraction to analyze the expression level of *ScWRKY3* in *N. benthamiana* by semi-quantitative PCR with the specific primer *ScWRKY3*-Gate-F/R (Table S2). The *NtEF-1α* (GenBank Accession No. D63396) gene was used as the reference gene. The semi-quantitative PCR program was set as: 94 °C, 4 min; 94 °C, 30 s; 65 °C, 30 s; 72 °C, 1 min plus 30 s; 35 cycles; and 72 °C, 10 min. Two important tobacco pathogens, including the bacterial pathogen of *R. solanacearum* and the fungal pathogen of *F. solani* var. *coeruleum*, were cultured overnight in potato dextrose water (PDW) liquid medium at 200 rpm and 28 °C. Then the two cultured pathogen cells were separately injected into the 1-day overexpressing *N. benthamiana* leaves after being diluted to OD_600_ = 0.6 using 10 mM magnesium chloride (MgCl_2_) solution. All treated plants were maintained for one week at 28 °C with a photoperiod of 16-h light/8-h darkness to track the changes in leaf symptoms and to analyze the relative transcript level of nine tobacco immunity-associated marker genes, including the HR marker genes *NtHSR201*, *NtHSR203*, and *NtHSR515*; the SA-related genes *NtPR-1a/c* and *NtNPR1*; the JA-associated genes *NtPR2* and *NtPR3*; and the ET synthesis-dependent genes *NtEFE26* and *NtAccdeaminase* (Tables S2 and S7–S9) [83,84]. All the treatments were carried out in three replicates. For representational observation, *Agrobacterium* suspensions carrying the vector pEarleyGate 203 and the recombinant vector pEarleyGate 203-*ScWRKY3* were injected into the left and right side of each selected *N. benthamiana* leaf, respectively.

## 5. Conclusions

In this study, a novel *ScWRKY3* gene was isolated from sugarcane and functionally characterized. ScWRKY3 belongs to group IIc of the WRKY family as a nucleoprotein, with no auto-activation. It has an interaction with another group IIc sugarcane WRKY protein, ScWRKY4, however the interaction mechanism and its corresponding function need further investigation. ScWRKY3 may participate in sugarcane resistance to drought and salt stimuli, and this resistance may be mediated by ABA signaling pathways. The transcript abundance of *ScWRKY3* was stable in the smut-resistant cultivar Yacheng05-179, while it was down-regulated in the smut-susceptible cultivar ROC22 at 24 h, during inoculation with *S. scitamineum*. In addition, *ScWRKY3* showed a negative regulatory effect on the bacterial pathogen *R. solanacearum* and the fungal disease *F. solani* var. *coeruleum* in *35S::ScWRKY3*-overexpressing *N. benthamiana*. These results may be useful for the functional identification of the WRKY family in sugarcane and good for the interaction analysis of ScWRKY3 with other WRKY proteins or other functional proteins.

## Supplementary Materials

Supplementary materials can be found at http://www.mdpi.com/1422-0067/19/12/4059/s1, **Figure S1**: Nucleic acid sequences alignment of *ScWRKY3* in ROC22 and Yacheng05-179; **Figure S2.** Secondary structure prediction of ScWRKY3; **Figure S3.** Signal peptide and transmembrane domain prediction of ScWRKY3; **Figure S4.** Subcellular localization prediction of ScWRKY3; **Figure S5.** Amino acid sequences alignment of ScWRKY3 and other WRKYs; **Figure S6.** The logo of predicted conserved motifs in the WRKYs; **Table S1.** Primary structure analysis of ScWRKY3; **Table S2.** Primers used in this study; **Table S3.** Raw calculations of tissue-specific expression of *ScWRKY3* in different 10-month-old ROC22 tissues by qRT-PCR; **Table S4.** Raw calculations of gene expression patterns of *ScWRKY3* in 4-month-old ROC22 plantlets under abiotic stress; **Table S5.** Raw calculations of gene expression of *ScWRKY3* in 4-month-old ROC22 plantlets under plant hormone stress; **Table S6.** Raw calculations of expression of the *ScWRKY3* gene after infection with smut pathogen; **Table S7.** Raw calculations of the transcript level of nine immunity-associated marker genes in the *Nicotiana benthamiana* leaves after one day of agroinfiltration; **Table S8.** Raw calculations of the transcripts of nine immunity-associated marker genes in the *Nicotiana benthamiana* leaves after inoculation with *Ralstonia solanacearum* for one day and seven days; **Table S9**. Raw calculations of the transcripts of nine immunity-associated marker genes in the *Nicotiana benthamiana* leaves after inoculation with *Fusarium solani* var. *coeruleum* for one day and seven days.

## References

[1] Ulker B., Somssich I.E. (2004). WRKY transcription factors: From DNA binding towards biological function. Curr. Opin. Plant Biol..

[2] Jiang J.J., Ma S.H., Ye N.H., Jiang M., Cao J.S., Zhang J.H. (2017). WRKY transcription factors in plant responses to stresses. J. Integr. Plant Biol..

[3] Singh K.B., Foley R.C., Oñatesánchez L. (2002). Transcription factors in plant defense and stress responses. Curr. Opin. Plant Biol..

[4] Chen L.G., Song Y., Li S.J., Li S.J., Zhang L.P., Zou C.S., Yu D.Q. (2012). The role of WRKY transcription factors in plant abiotic stresses. Biochim. Biophys. Acta.

[5] Rushton P.J., Somssich I.E., Ringler P., Shen Q.J. (2010). WRKY transcription factors. Trends Plant Sci..

[6] Eulgem T., Rushton P.J., Robatzek S., Somssich I.E. (2000). The WRKY superfamily of plant transcription factors. Trends Plant Sci..

[7] Mohanta T.K., Park Y.H., Bae H. (2016). Novel genomic and evolutionary insight of WRKY transcription factors in plant lineage. Sci. Rep..

[8] Chen F., Hu Y., Vannozzi A., Wu K.C., Cai H.Y., Qin Y., Mullis A., Lin Z.G., Zhang L.S. (2018). The WRKY transcription factor family in model plants and crops. Plant Sci..

[9] Ramamoorthy R., Jiang S.Y., Kumar N., Venkatesh P.N., Ramachandran S. (2008). A comprehensive transcriptional profiling of the *WRKY* gene family in rice under various abiotic and phytohormone treatments. Plant. Cell Physiol..

[10] Wei K., Chen J., Chen Y.F., Wu L.J., Xie D.X. (2012). Multiple-strategy analyses of ZmWRKY subgroups and functional exploration of *ZmWRKY* genes in pathogen responses. Mol. Biosyst..

[11] Muthamilarasan M., Bonthala V.S., Khandelwal R., Jaishankar J., Shweta S., Nawaz K., Prasad M. (2015). Global analysis of WRKY transcription factor superfamily in *Setaria* identifies potential candidates involved in abiotic stress signaling. Front. Plant Sci..

[12] Pandey S.P., Somssich I.E. (2009). The role of WRKY transcription factors in plant immunity. Plant Physiol..

[13] Mangelsen E., Kilian J., Berendzen K.W., Kolukisaoglu U.H., Harter K., Jansson C., Wanke D. (2008). Phylogenetic and comparative gene expression analysis of barley (*Hordeum vulgare*) WRKY transcription factor family reveals putatively retained functions between monocots and dicots. BMC Genomics.

[14] Wei X.A., Yao W.J., Jiang T.B., Zhou B.R. (2016). Identification of *WRKY* gene in response to abiotic stress from WRKY transcirption factor gene family of *Arabidopsis thaliana*. J. Northeast. For. Univ..

[15] Zhang T., Tan D., Zhang L., Zhang X., Han Z. (2017). Phylogenetic analysis and drought-responsive expression profiles of the WRKY transcription factor family in maize. Agric. Gene.

[16] Wu J., Chen J.B., Wang L.F., Wang S.M. (2017). Genome-wide investigation of WRKY transcription factors involved in terminal drought stress response in common bean. Front. Plant Sci..

[17] Qiu Y.Q., Jing S.J., Fu J., Li L., Yu D.Q. (2004). Cloning and analysis of expression profile of 13 *WRKY* genes in rice. Chin. Sci. Bull..

[18] Wu H.L., Ni Z.F., Yao Y.Y., Guo G.G., Sun Q.X. (2008). Cloning and expression profiles of 15 genes encoding WRKY transcription factor in wheat (*Triticum aestivem* L.). Prog. Nat. Sci. Mater. Int..

[19] Dong J., Chen C.H., Chen Z.X. (2003). Expression profiles of the *Arabidopsis WRKY* gene superfamily during plant defense response. Plant Mol. Biol..

[20] Kim K.C., Lai Z., Fan B., Chen Z. (2008). *Arabidopsis* WRKY38 and WRKY62 transcription factors interact with histone deacetylase 19 in basal defense. Plant Cell.

[21] Liu D.L., Leib K., Zhao P.Y., Kogel K.H., Langen G. (2014). Phylogenetic analysis of barley WRKY proteins and characterization of HvWRKY1 and repressors of the pathogen-inducible gene *HvGER4c*. Mol. Genet. Genomics.

[22] Peng Y., Bartley L.E., Chen X.W., Dardick C., Chern M., Ruan R., Canlas P.E., Ronald P.C. (2008). *OsWRKY62* is a negative regulator of basal and *Xa21*-mediated defense against *Xanthomonas orvzae* pv. *orvzae* in rice. Mol. Plant.

[23] Berri S., Abbruscato P., Faivre-Rampant O., Brasileiro A.C., Fumasoni I., Satoh K., Kikuchi S., Mizzi L., Morandini P., Pè M.E. (2009). Characterization of WRKY co-regulatory networks in rice and *Arabidopsis*. BMC Plant Biol..

[24] Banerjee A., Roychoudhury A. (2015). WRKY proteins: Signaling and regulation of expression during abiotic stress responses. Sci. World J..

[25] Chen R.K., Xu L.P., Lin Y.Q. (2011). Modern Sugarcane Genetic Breeding.

[26] Lambais M.R. (2001). *In silico* differential display of defense-related expressed sequence tags from sugarcane tissues infected with *Diazotrophic endophytes*. Genet. Mol. Biol..

[27] Liu J.X., Que Y.X., Guo J.L., Xu L.P., Wu J.Y., Chen R.K. (2012). Molecular cloning and expression analysis of a WRKY transcription factor in sugarcane. Afr. J. Biotechnol..

[28] Wang L., Liu F., Dai M.J., Sun T.T., Su W.H., Wang C.F., Zhang X., Mao H.Y., Su Y.C., Que Y.X. (2018). Cloning and expression characteristic analysis of *ScWRKY4* gene in sugarcane. Acta Agron. Sin..

[29] Que Y.X., Su Y.C., Guo J.L., Wu Q.B., Xu L.P. (2014). A global view of transcriptome dynamics during *Sporisorium scitamineum* challenge in sugarcane by RNAseq. PLoS ONE.

[30] Chou K.C., Shen H.B. (2010). A new method for predicting the subcellular localization of eukaryotic proteins with both single and multiple sites: Euk-mPLoc 2.0. PLoS ONE.

[31] Ishiguro S., Nakamura K. (1994). Characterization of a cDNA encoding a novel DNA-binding protein, *SPF1*, that recognizes SP8 sequences in the 5′ upstream regions of genes coding for sporamin and beta-amylase from sweet potato. Mol. Gen. Genet..

[32] Eulgem T., Rushton P.J., Schmelzer E., Hahlbrock K., Somssich I.E. (1999). Early nuclear events in plant defence signalling: Rapid gene activation by WRKY transcription factors. Embo J..

[33] Liu L.S., White M.J., Macrae T. (2010). Transcription factors and their genes in higher plants. FEBS J..

[34] Fan Z.Q., Tan X.L., Shan W., Kuang J.F., Lu W.J., Chen J.Y. (2017). BrWRKY65, a WRKY transcription factor, is involved in regulating three leaf senescence-associated genes in Chinese flowering cabbage. Int. J. Mol. Sci..

[35] Wan Y.Q., Mao M.Z., Wan D.L., Yang Q., Yang F.Y., Mandlaa, Li G.J., Wang R.G. (2018). Identification of the *WRKY* gene family and functional analysis of two genes in *Caragana intermedia*. BMC Plant Biol..

[36] Lee H., Cha J., Choi C., Choi N., Ji H.S., Park S.R., Lee S., Hwang D.J. (2018). Rice *WRKY11* plays a role in pathogen defense and drought tolerance. Rice.

[37] Wang H.H., Meng J., Peng X.X., Tang X.K., Zhou P.L., Xiang J.H., Deng X.B. (2015). Rice *WRKY4* acts as a transcriptional activator mediating defense responses toward *Rhizoctonia solani*, the causing agent of rice sheath blight. Plant Mol. Biol..

[38] Fields S., Song O. (1989). A novel genetic system to detect protein-protein interactions. Nature.

[39] Sébastien B., Li J., Palva E.T. (2012). WRKY54 and WRKY70 co-operate as negative regulators of leaf senescence in *Arabidopsis thaliana*. J. Exp. Bot..

[40] Wang X.T., Zeng J., Li Y., Rong X.L., Sun J.T., Sun T., Li M., Wang L.Z., Feng Y., Chai R.H. (2015). Expression of *TaWRKY44*, a wheat *WRKY* gene, in transgenic tobacco confers multiple abiotic stress tolerances. Front. Plant Sci..

[41] Chi Y.C., Yang Y., Zhou Y., Zhou J., Fan B.F., Yu J.Q., Chen Z.X. (2013). Protein-protein interactions in the regulation of WRKY transcription factors. Mol. Plant.

[42] Robatzek S., Somssich I.E. (2002). Targets of *AtWRKY6* regulation during plant senescence and pathogen defense. Genes Dev..

[43] Popescu S.C., Popeseu G.V., Bachan S., Zhang Z., Gerstein M., Snyder M., Dinesh-Kumar S.E. (2009). MAPK target networks in *Arabidopsis thaliana* revealed using functional protein microarrays. Genes Dev..

[44] Zhou L., Wang N.N., Kong L., Gong S.Y., Li Y., Li X.B. (2014). Molecular characterization of 26 cotton *WRKY* genes that are expressed differentially in tissues and are induced in seedlings under high salinity and osmotic stress. Plant Cell Tissue Org. Cult..

[45] Bakshi M., Oelmüller R. (2014). WRKY transcription factors: Jack of many trades in plants. Plant Signal. Behav..

[46] Bartels D., Sunkar R. (2005). Drought and salt tolerance in plants. Crit. Rev. Plant Sci..

[47] Qiao Z., Li C.L., Zhang W. (2016). *WRKY1* regulates stomatal movement in drought-stressed *Arabidopsis thaliana*. Plant Mol. Biol..

[48] Jiang Y.J., Yu D.Q. (2016). *WRKY57* regulates *JAZ* genes transcriptionally to compromise *Botrytis cinerea* resistance in *Arabidopsis thaliana*. Plant Physiol..

[49] Jones J.D., Dang J.L. (2006). The plant immune system. Nature.

[50] Métraux J.P., Nawrath C., Genoud T. (2002). Systemic acquired resistance. Euphytica.

[51] Katagiri F. (2004). A global view of defense gene expression regulation—A highly interconnected signaling network. Curr. Opin. Plant Biol..

[52] Kunkel B.N., Brooks D.M. (2002). Cross talk between signaling pathways in pathogen defense. Curr. Opin. Plant Biol..

[53] Chen X.T., Liu J., Lin G.F., Wang A., Wang Z.G., Lu G.D. (2013). Overexpression of *AtWRKY28* and *AtWRKY75* in *Arabidopsis* enhances resistance to oxalic acid and *Sclerotinia sclerotiorum*. Plant Cell Rep..

[54] Wang H.H., Hao J.J., Chen X.J., Hao Z.N., Wang X., Lou Y.G., Peng Y.L., Guo Z.J. (2007). Overexpression of rice *WRKY89* enhances ultraviolet B tolerance and disease resistance in rice plants. Plant Mol. Biol..

[55] Qiu D.Y., Xiao J., Ding X.H., Xiong M., Cai M., Cao Y.L., Li X.H., Xu C.G., Wang S.P. (2007). *OsWRKY13* mediates rice disease resistance by regulating defense-related genes in salicylate- and jasmonate-dependent signaling. Mol. Plant-Microbe Interact..

[56] Qiu D.Y., Xiao J., Xie W.B., Liu H.B., Li X.H., Xiong L.Z., Wang S.P. (2008). Rice gene network inferred from expression profiling of plants overexpressing *OsWRKY13*, a positive regulator of disease resistance. Mol. Plant.

[57] Zheng Z.Y., Mosher S.L., Fan B.F., Klessig D.F., Chen Z.X. (2007). Functional analysis of *Arabidopsis* WRKY25 transcription factor in plant defense against *Pseudomonas syringae*. BMC Plant Biol..

[58] Jiang Y., Deyholos M.K. (2009). Functional characterization of *Arabidopsis* NaCl-inducible WRKY25 and WRKY33 transcription factors in abiotic stresses. Plant Mol. Biol..

[59] Yokotani N., Sato Y., Tanabe S., Chujo T., Shimizu T., Okada K., Yamane K., Shimono M., Sugano S., Takatsuji H. (2013). WRKY76 is a rice transcriptional repressor playing opposite roles in blast disease resistance and cold stress tolerance. J. Exp. Bot..

[60] Liu X.F., Song Y.Z., Xing F.Y., Wang N., Wen F.J. (2015). *GhWRKY25*, a group I *WRKY* gene from cotton, confers differential tolerance to abiotic and biotic stresses in transgenic *Nicotiana benthamiana*. Protoplasma.

[61] Wang X., Yan Y., Li Y.Z., Chu X.Q., Wu C.G., Guo X.Q. (2014). *GhWRKY40*, a multiple stress-responsive cotton *WRKY* gene, plays an important role in the wounding response and enhances susceptibility to *Ralstonia solanacearum* infection in transgenic *Nicotiana benthamiana*. PLoS ONE.

[62] Mukhtar M.S., Deslandes L., Auriac M., Marco Y., Somssich I.E. (2008). The Arabidopsis transcription factor WRKY27 influences wilt disease symptom development caused by *Ralstonia solanacearum*. Plant J..

[63] Su Y.C., Guo J.L., Ling H., Chen S.S., Wang S.S., Xu L.P., Allan A.C., Que Y.X. (2014). Isolation of a novel peroxisomal catalase gene from sugarcane, which is responsive to biotic and abiotic stresses. PLoS ONE.

[64] Li H., Gao Y., Xu H., Dai Y., Deng D.Q., Chen J.M. (2013). *ZmWRKY33*, a WRKY maize transcription factor conferring enhanced salt stress tolerances in *Arabidopsis*. Plant Growth Regul..

[65] Scarpeci T.E., Zanor M.I., Zanor M.I., Mueller-Roeber B., Valle E.M. (2013). Overexpression of *AtWRKY30* enhances abiotic stress tolerance during early growth stages in *Arabidopsis thaliana*. Plant Mol. Biol..

[66] Marchlerbauer A., Bo Y., Han L.Y., He J., Lanczycki C.J., Lu S.N., Chitsaz F., Derbyshire M.K., Geer R.C., Gonzales N.R. (2017). CDD/SPARCLE: Functional classification of proteins via subfamily domain architectures. Nucleic Acids Res..

[67] Gasteiger E., Hoogland C., Gattiker A., Duvaud S., Wilkins M.R., Appel R.D., Bairoch A. (2005). Protein identification and analysis tools on the ExPASy server. The Proteomics Protocols Handbook.

[68] Combet C., Blanchet C., Geourjon C., Deléage G. (2000). NPS@: Network protein sequence analysis. Trends Biochem. Sci..

[69] Petersen T.N., Brunak S., Heijne G., Nielsen H. (2011). SignalP 4.0: Discriminating signal peptides from transmembrane regions. Nat. Methods.

[70] Nielsen H. (2017). Predicting secretory proteins with SignalP. Methods Mol. Biol..

[71] Krogh A., Larsson B., Heijne G.V., Sonnhammer E.L.L. (2001). Predicting transmembrane protein topology with a hidden markov model: Application to complete genomes. J. Mol. Biol..

[72] Kumar S., Stecher G., Tamura K. (2016). MEGA7: Molecular evolutionary genetics analysis version 7.0 for bigger datasets. Mol. Biol. Evol..

[73] Wang C., Deng P., Chen L., Wang X., Ma H., Hu W., Yao N.C., Feng Y., Chai R.H., Yang G.X. (2013). A wheat WRKY transcription factor TaWRKY10 confers tolerance to multiple abiotic stresses in transgenic tobacco. PLoS ONE.

[74] Curtis M.D., Grossniklaus U. (2003). A gateway cloning vector set for high-throughput functional analysis of genes in planta. Plant Phyosiol..

[75] Hwang I.S., Hwang B.K. (2012). Requirement of the cytosolic interaction between pathogenesis-related protein10 and leucine-rich repeat protein1 for cell death and defense signaling in pepper. Plant Cell.

[76] Fan Z.Q., Kuang J.F., Fu C.C., Shan W., Han Y.C., Xiao Y.Y., Ye Y.J., Lu W.J., Lakshmanan P., Duan X.W. (2016). The banana transcriptional repressor *MaDEAR1* negatively regulates cell wall-modifying genes involved in fruit ripening. Front. Plant Sci..

[77] Shen C.J., Wang S.K., Bai Y.H., Wu Y.R., Zhang S.N., Chen M., Guilfoyle T.J., Wu P., Qi Y.H. (2010). Functional analysis of the structural domain of ARF proteins in rice (*Oryza sativa* L.). J. Exp. Bot..

[78] Schütze K., Harter K., Chaban C. (2009). Bimolecular fluorescence complementation (BiFC) to study protein-protein interactions in living plant cells. Methods Mol. Biol..

[79] Mersereau M., Pazour G.J., Das A. (1990). Efficient transformation of *Agrobacterium tumefaciens* by electroporation. Gene.

[80] Livak K.J., Schmittgen T.D. (2001). Analysis of relative gene expression data using real-time quantitative PCR and the 2^−∆∆*C*^_T_ method. Methods.

[81] Su Y.C., Wang Z.Q., Li Z., Xu L.P., Que Y.X., Dai M.J., Chen Y.H. (2017). Molecular cloning and functional identification of peroxidase gene *ScPOD02* in sugarcane. Acta Agron. Sin..

[82] Earley K.W., Haag J.R., Pontes O., Opper K., Juehne S., Song K., Pikaard C.S. (2006). Gateway-compatible vectors for plant functional genomics and proteomics. Plant J..

[83] Peng Q., Su Y.C., Ling H., Ahmad W., Gao S.W., Guo J.L., Que Y.X., Xu L.P. (2017). A sugarcane pathogenesis-related protein, *ScPR10*, plays a positive role in defense responses under *Sporisorium scitamineum*, SrMV, SA, and MeJA stresses. Plant Cell Rep..

[84] Lai Y., Dang F.F., Lin J., Yu L., Shi Y.L., Xiao Y.H., Huang M.K., Lin J.H., Chen C.C., Qi A.H. (2013). Overexpression of a *Chinese cabbage BrERF11*, transcription factor enhances disease resistance to *Ralstonia solanacearum*, in *tobacco*. Plant Physiol. Biochem..

