# Supplementary material for: Expression Characteristics and Functional Analysis of the ScWRKY3 Gene from Sugarcane
Source: Int J Mol Sci. 2018 Dec 14;19(12):4059. doi: 10.3390/ijms19124059 (PMC6321069; doi:10.3390/ijms19124059)
Supplement: Supplementary file 1 [file ijms-19-04059-s001.pdf]

International Journal of
Molecular Sciences

MDPI

Article

Expression Characteristics and Functional Analysis of the *ScWRKY3* Gene from Sugarcane

Ling Wang [1], Feng Liu [1], Xu Zhang [1], Wenju Wang [1], Tingting Sun [1], Yufeng Chen [1], Mingjian Dai [1], Shengxiao Yu [1], Liping Xu [1,2], Yachun Su [1,2,*] and Youxiong Que [1,2,*]

[1] Key Laboratory of Sugarcane Biology and Genetic Breeding, Ministry of Agriculture, Fujian Agriculture and Forestry University, Fuzhou 350002, China; lingw2017@126.com (L.W.); 18359162091@163.com (F.L.); zahngxuqq7@126.com (X.Z.); wwj1470665850@163.com (W.W.); sunting3221@163.com (T.S.); CYF9410@163.com (Y.C.); 18906071567@163.com (M.D.); dbzq666666@163.com (S.Y.); xlpmail@126.com (L.X.);

[2] Key Laboratory of Ministry of Education for Genetics, Breeding and Multiple Utilization of Crops, College of Crop Science, Fujian Agriculture and Forestry University, Fuzhou 350002, China

* Correspondence: syc2009mail@163.com, queyouxiong@126.com; Tel.: +86-591-8385-2547

Supplementary Materials

Table S1. Primary structure analysis of ScWRKY3

**Primary structure characteristics**	**Prediction results**
Number of amino acids	236
Molecular weight	25979.45 Da
Theoretical isoelectric point (pI)	8.58
Grand average of hydropathicity (GRAVY)	-0.492
The instability index (II)	56.08

The primary structure of the ScWRKY3 protein was predicted by ProtParam (https://web.expasy.org/protparam/).

Table S2. Primers used in this study

**Primer name**	**Primer sequence (5′-3′)**	**Usage**
ScWRKY3-F	ACCACCACTAACCCCAAAGC	Full length amplification
ScWRKY3-R	CTGCGTCGTCAATGTATGCG	Full length amplification
ScWRKY3-QF	GCGACGTGGATGTACTGGATGA	qRT-PCR analysis
ScWRKY3-QR	CCTTGGATGGAGGCTGTTCTTG	qRT-PCR analysis
GAPDH-F	CACGGCCACTGGAAGCA	qRT-PCR analysis
GAPDH-R	TCCTCAGGGTTCCTGATGCC	qRT-PCR analysis
ScWRKY3-Gate-F	GGGGACAAGTTTGTACAAAAAAGCAGGCTTCAT GCAGGCATATATGGAGGG	Gateway entry vector construction and semi-quantitative PCR analysis
ScWRKY3-Gate-R	GGGGACCACTTTGTACAAGAAAGCTGGGTCGAA GGAGCTGAAGCAATCGG	Gateway entry vector construction and semi-quantitative PCR analysis
ScWRKY4-Gate-F	GGGGACAAGTTTGTACAAAAAAGCAGGCTTCAT GGAGGGGAGCAGCCAGCT	Gateway entry vector construction
ScWRKY4-Gate-R	GGGGACCACTTTGTACAAGAAAGCTGGGTCGAG CGACGTGAAAGCGCAGC	Gateway entry vector construction
ScWRKY3-BD-F	GGAATTCCATATGATGCAGGCATATATGGAGGG	Bait vector construction
ScWRKY3-BD-R	CGGGATCCGAAGGAGCTGAAGCAATCGG	Bait vector construction
ScWRKY3-AD-F	GGAATTCCATATGATGCAGGCATATATGGAGGG	Prey vector construction
ScWRKY3-AD-R	CGGGATCCGAAGGAGCTGAAGCAATCGG	Prey vector construction
ScWRKY4-BD-F	GGAATTCCATATGATGGAGGGGAGCAGCCAGCT	Bait vector construction
ScWRKY4-BD-R	CGGGATCCGAGCGACGTGAAAGCGCAGC	Bait vector construction
ScWRKY4-AD-F	CCGGAATTCATGGAGGGGAGCAGCCAGCT	Prey vector construction
ScWRKY4-AD-R	CCGCTCGAGGAGCGACGTGAAAGCGCAGC	Prey vector construction
NtH201-F	CAGCAGTCCTTTGGCGTTGTC	qRT-PCR analysis
NtH201-R	GCTCAGTTTAGCCGCAGTTGTG	qRT-PCR analysis
NtH203-F	TGGCTCAACGATTACGCA	qRT-PCR analysis
NtH203-R	GCACGAAACCTGGATGG	qRT-PCR analysis
NtH515-F	TTGGGCAGAATAGATGGGTA	qRT-PCR analysis
NtH515-R	TTTGGTGAAAGTCTTGGCTC	qRT-PCR analysis
NtNPR1-F	GGCGAGGAGTCCGTTCTTTAA	qRT-PCR analysis
NtNPR1-R	TCAACCAGGAATGCCACAGC	qRT-PCR analysis
NtPR1a/c-F	AACCTTTGACCTGGGACGAC	qRT-PCR analysis
NtPR1a/c-R	GCACATCCAACACGAACCGA	qRT-PCR analysis
NtPR2-F	TGATGCCCTTTTGGATTCTATG	qRT-PCR analysis
NtPR2-R	AGTTCCTGCCCCGCTTT	qRT-PCR analysis
NtPR3-F	CAGGAGGGTATTGCTTTGTTAGG	qRT-PCR analysis
NtPR3-R	CGTGGGAAGATGGCTTGTTGTC	qRT-PCR analysis
NtEFE26-F	CGGACGCTGGTGGCATAAT	qRT-PCR analysis
NtEFE26-R	CAACAAGAGCTGGTGCTGGATA	qRT-PCR analysis
NtAccdeaminase-F	TCTGAGGTTACTGATTTGGATTGG	qRT-PCR analysis
NtAccdeaminase-R	TGGACATGGTGGATAGTTGCT	qRT-PCR analysis
NtEF-1α-F	TGCTGCTGTAACAAGATGGATGC	qRT-PCR analysis
NtEF-1α-R	GAGATGGGGACAAAGGGGATT	qRT-PCR analysis

attB1 and *attB2* adapters are underlined in the forward primers and in the reverse primers for gateway entry vector construction, respectively. The added restriction enzyme sites of *Eco*R I (GAATTC), *Xho* I (CTCGAG), *Nde* I (CATATG)

and *Bam*H I (GGATCC) are underlined in the primers for bait or prey vectors construction, and the double underlined areas in these primers indicate protective bases.

Table S3. Raw calculations of tissue-specific expression of *ScWRKY3* in different 10-month-old ROC22 tissues by qRT-PCR

Tissues	$2^{-\Delta\Delta C_T}$	Standard error
R	1.02874	0.17959
B	4.70889	1.64113
L	1.13441	0.35043
SP	29.17377	4.75581
SE	2.50973	0.36618

The tissues (root, bud, leaf, stem pith, and stem epidermis) are represented by R, B, L, SP, and SE, respectively. Data are normalized to the glyceraldehyde-3-phosphate dehydrogenase (*GAPDH*) expression level. All data points are means ± standard error (*n* = *3*). Bars superscripted by different lowercase letters indicate significant differences, as determined by Duncan's new multiple range test (p-value < 0.05).

Table S4. Raw calculations of gene expression patterns of *ScWRKY3* in 4-month-old ROC22 plantlets under abiotic stress

Treatment time	NaCl		PEG	
	$2^{-\Delta\Delta C_T}$	Standard error	$2^{-\Delta\Delta C_T}$	Standard error
0 h	1.02248	0.15162	1.00235	0.04850
0.5 h	1.12695	0.03042	0.67118	0.05745
3 h	1.10801	0.06703	38.57182	10.9277
6 h	0.63938	0.08744	1.27614	0.22401
24 h	3.27612	0.26971	1.11389	0.12550

NaCl, sodium chloride (simulating salt stress) (250 mM); PEG, polyethylene glycol (simulating drought treatment) (25.0%). Data are normalized to the glyceraldehyde-3-phosphate dehydrogenase (*GAPDH*) expression level. All data points are means ± standard error (*n* = *3*). Bars superscripted by different lowercase letters indicate significant differences, as determined by Duncan's new multiple range test (p-value < 0.05).

Table S5. Raw calculations of gene expression of *ScWRKY3* in 4-month-old ROC22 plantlets under plant hormone stress

Treatment time	SA		MeJA		ABA	
	$2^{-\Delta\Delta C_T}$	Standard error	$2^{-\Delta\Delta C_T}$	Standard error	$2^{-\Delta\Delta C_T}$	Standard error
0 h	1.00130	0.00001	1.00217	0.00011	1.00515	0.08640
3 h	0.39460	0.01486	0.55133	0.00048	0.80955	0.04149
6 h	0.59523	0.10924	0.42110	0.05784	1.66832	0.15545
24 h	0.49914	0.00962	0.33474	0.08544	0.80563	0.11352

SA, salicylic acid (5 mM); MeJA, methyl jasmonate (25 μM); ABA, abscisic acid (100 μM). Data are normalized to the glyceraldehyde-3-phosphate dehydrogenase (*GAPDH*) expression level. All data points are means ± standard error (*n* = *3*). Bars superscripted by different lowercase letters indicate significant differences, as determined by Duncan's new multiple range test (p-value < 0.05).

Table S6. Raw calculations of expression of the *ScWRKY3* gene after infection with smut pathogen

Treatment time	Yacheng05-179		ROC22	
	$2^{-\Delta\Delta C_T}$	Standard error	$2^{-\Delta\Delta C_T}$	Standard error
0 h	1.02011	0.25922	1.00740	0.08826
24 h	1.26167	0.47474	0.50795	0.06088
48 h	1.13023	0.09219	0.95991	0.00450
72 h	1.05532	0.10303	0.79783	0.03618

Yacheng05-179 is a smut-resistant *Saccharum* hybrid cultivar, and ROC22 is a smut-susceptible *Saccharum* hybrid cultivar. Data are normalized to the glyceraldehyde-3-phosphate dehydrogenase (*GAPDH*) expression level. All data points are means ± standard error (*n* = *3*). Bars superscripted by different lowercase letters indicate significant differences, as determined by Duncan's new multiple range test (p-value < 0.05).

Table S7. Raw calculations of the transcript level of nine immunity-associated marker genes in the *Nicotiana benthamiana* leaves after one day of agroinfiltration

Gene name	*35::00*		*35::ScWRKY3*	
	$2^{-\Delta\Delta C_T}$	Standard error	$2^{-\Delta\Delta C_T}$	Standard error
NtHSR201	1.00035	0.01878	0.31325	0.01010
NtHSR203	1.02856	0.16092	5.55995	0.26401
NtHSR515	1.01574	0.12341	3.05449	0.03158
NtPR1	1.02067	0.15147	14.15632	0.80030
NtPR-1a/c	1.00025	0.01561	0.83799	0.01543
NtPR2	1.00014	0.01178	0.99941	0.02160
NtPR3	1.00096	0.03072	8.79463	0.34939
NtEFE26	1.00027	0.01638	1.46628	0.01135
NtAccdeaminase	1.00442	0.06553	2.35996	0.06627

Data are normalized to the *NtEF-1α* expression level. All data points are means ± standard error (*n* = *3*). Bars superscripted by different lowercase letters indicate significant differences, as determined by Duncan's new multiple range test (p-value < 0.05).

Table S8. Raw calculations of the transcripts of nine immunity-associated marker genes in the *Nicotiana benthamiana* leaves after inoculation with *Ralstonia solanacearum* for one day and seven days

Gene name	Treatment time	*35::00*		*35::ScWRKY3*	
		$2^{-\Delta\Delta C_T}$	Standard error	$2^{-\Delta\Delta C_T}$	Standard error
NtH201	0 d	1.00035	0.01878	1.03671	0.18533
	1 d	23.83326	0.44415	1.00248	0.05525
	7 d	21.64681	0.79454	1.00289	0.04423
NtH203	0 d	1.02856	0.16092	1.00226	0.04759
	1 d	8.47359	0.08132	2.51027	0.04509
	7 d	1.13105	0.03165	3.32439	0.10266
NtH515	0 d	1.01574	0.12341	1.00011	0.01034
	1 d	23.12115	0.75118	2.70385	0.15356
	7 d	7.13494	0.12217	2.65545	0.04517
NtNPR1	0 d	1.02067	0.15147	1.00310	0.05671
	1 d	48.32176	3.78460	24.73383	3.91994
	7 d	26.22899	0.03695	12.70940	1.69010
NtPR1a/c	0 d	1.00025	0.01561	1.00087	0.02918
	1 d	2.69564	0.19104	2.23772	0.04028
	7 d	7.34399	0.43130	1.03147	0.03602
NtPR2	0 d	1.00014	0.01178	1.43645	0.85614
	1 d	0.00540	0.00130	0.00003	0.00001
	7 d	6.91943	1.35432	5.07464	0.76171
NtPR3	0 d	1.00096	0.03072	1.00158	0.03979
	1 d	1.17475	0.02933	0.78957	0.29541
	7 d	4.22494	0.65946	1.95949	0.16057
NtEFE26	0 d	1.00027	0.01638	1.00006	0.00774
	1 d	0.80520	0.01612	0.29558	0.00401
	7 d	0.20565	0.00213	0.07477	0.00183
NtAccdeaminase	0 d	1.00442	0.06553	1.00078	0.02811
	1 d	0.60119	0.01118	0.63861	0.02083
	7 d	0.29504	0.01311	0.63590	0.00549

Data are normalized to the *NtEF-1α* expression level. All data points are means ± standard error ($n = 3$). Bars superscripted by different lowercase letters indicate significant differences, as determined by Duncan's new multiple range test (p-value < 0.05).

Table S9. Raw calculations of the transcripts of nine immunity-associated marker genes in the *Nicotiana benthamiana* leaves after inoculation with *Fusarium solani* var. *coeruleum* for one day and seven days

**Gene name**	**Treatment time**	***35::00***		***35::ScWRKY3***	
		**$2^{-\Delta\Delta C_T}$**	**Standard error**	**$2^{-\Delta\Delta C_T}$**	**Standard error**
NtH201	0 d	1.00195	0.04430	1.00045	0.02137
	1 d	0.27135	0.01817	0.53091	0.01105
	7 d	0.05003	0.00352	0.14749	0.00262
NtH203	0 d	1.00208	0.04575	1.00036	0.01894
	1 d	0.67138	0.03458	0.42983	0.03520
	7 d	0.11990	0.00492	0.26738	0.00426
NtH515	0 d	1.01527	0.11926	1.02883	0.16165
	1 d	13.54125	0.28097	0.00261	0.00001
	7 d	1.19530	0.00971	0.00024	0.00002
NtNPR1	0 d	1.00195	0.05101	1.02043	0.16589
	1 d	6.91331	0.45229	1.99066	0.30924
	7 d	3.44026	0.03078	0.20613	0.02542
NtPR1a/c	0 d	1.00025	0.01561	1.00054	0.02319
	1 d	0.22801	0.04156	0.10524	0.00300
	7 d	0.99548	0.02271	0.27953	0.00774
NtPR2	0 d	1.00542	0.07383	1.02190	0.14310
	1 d	1.40590	0.24153	1.22961	0.13109
	7 d	0.04811	0.00295	0.64970	0.12044
NtPR3	0 d	1.00096	0.03072	1.00158	0.03979
	1 d	0.04353	0.00743	0.27462	0.03474
	7 d	0.80819	0.09846	2.35022	0.14629
NtEFE26	0 d	1.00077	0.02752	1.00006	0.00774
	1 d	0.00036	0.00002	0.00126	0.00006
	7 d	0.00021	0.02002	0.00032	0.00004
NtAccdeaminase	0 d	1.00442	0.06553	1.00078	0.02811
	1 d	0.13969	0.03101	1.68063	0.04270
	7 d	0.04792	0.00090	0.15812	0.03980

Data are normalized to the *NtEF-1α* expression level. All data points are means ± standard error ($n = 3$). Bars superscripted by different lowercase letters indicate significant differences, as determined by Duncan's new multiple range test (p-value < 0.05).

```
ROC22-ScWRKY3      ACCACCACTAACCCCAAAGCATCATCGTCATCGTCAACCTTATCGTGCACAGTTATTGACACCAAAAGGAGAGAGATGCA   80
YC05-179-ScWRKY3   ACCACCACTAACCCCAAAGCATCATCGTCATCGTCAACCTTATCGTGCACAGTTATTGACACCAAAAGGAGAGAGATGCA   80
Consensus          accaccactaaccccaaagcatcatcgtcatcgtcaaccttatcgtgcacagttattgacaccaaaaggagagagatgca

ROC22-ScWRKY3      TTCCGATCCATCCTGCGAATTTTCTGCCCCGGCCGCCCAACCAAGATCCACACTTTGATCATCAATCGATCGCTAGCGCA  160
YC05-179-ScWRKY3   TTCCGATCCATCCTGCGAATTTTCTGCCCCGGCCGCCCAACCAAGATCCACACTTTGATCATCAATCGATCGCTAGCGCA  160
Consensus          ttccgatccatcctgcgaattttctgccccggccgcccaaccaagatccacactttgatcatcaatcgatcgctagcgca

ROC22-ScWRKY3      TATGCAGGCATATATGGAGGGAGGCCAGTTGAGTGCTTGCCTTCCTAGCTTCCTTGTGCCGGATCACTACGCCGGCTTCC  240
YC05-179-ScWRKY3   TATGCAGGCATATATGGAGGGAGGCCAGTTGAGTGCTTGCCTTCCTAGCTTCCTTGTGCCGGATCACTACGCCGGCTTCC  240
Consensus          tatgcaggcatatatggagggaggccagttgagtgcttgccttcctagcttccttgtgccggatcactacgccggcttcc

ROC22-ScWRKY3      CCCTTCCTCTCCCGCTACAACTTCCTAGCCAACCAAACAACAAGCTTTTCCAGATGCCGTTTGTAGTTAACCAGGAAGAG  320
YC05-179-ScWRKY3   CCCTTCCTCTCCCGCTACAACTTCCTAGCCAACCAAACAACAAGCTTTTCCAGATGCCGTTTGTAGTTAACCAGGAAGAG  320
Consensus          cccttcctctcccgctacaacttcctagccaaccaaacaacaagcttttccagatgccgtttgtagttaaccaggaagag

ROC22-ScWRKY3      ACAGAGAACCATGGCGGCATGCTCTCCTCCGACCATTGTGGTGGACTATACCCGCTGCCGGCACTGCCCTTCGGCAGCTG  400
YC05-179-ScWRKY3   ACAGAGAACCATGGCGGCATGCTCTCCTCCGACCATTGTGGTGGACTATACCCGCTGCCGGCACTGCCCTTCGGCAGCTG  400
Consensus          acagagaaccatggcggcatgctctcctccgaccattgtggtggactatacccgctgccggcactgcccttcggcagctg

ROC22-ScWRKY3      CTCCGGCGCCGCCGCCGCAACAGCATGCGGTGGGAAGCCGACGGCCGGTTTCATGCCCAGTGCTATTGGCGCTGAGGAGG  480
YC05-179-ScWRKY3   CTCCGGCGCCGCCGCCGCAACAGCATGCGGTGGGAAGCCGACGGCCGGTTTCATGCCCAGTGCTATTGGCGCTGAGGAGG  480
Consensus          ctccggcgccgccgccgcaacagcatgcggtgggaagccgacggccggtttcatgcccagtgctattggcgctgaggagg

ROC22-ScWRKY3      TCTGCACCTCGGTGGCTACTAAACTAGGTTGCAACGAGAGTAATAGCACATGGTGGAAGGGCTCAGCAGCTACGACTGCG  560
YC05-179-ScWRKY3   TCTGCACCTCGGTGGCTACTAAACTAGGTTGCAACGAGAGTAATAGCACATGGTGGAAGGGCTCAGCAGCTACGACTGCG  560
Consensus          tctgcacctcggtggctactaaactaggttgcaacgagagtaatagcacatggtggaagggctcagcagctacgactgcg

ROC22-ScWRKY3      GAGAGAGGGAAGATGAAGGTGAGGAGGAAGATGAGGGAACCGAGGTTTTGCTTCCAGACCAGAAGCGACGTGGATGTACT  640
YC05-179-ScWRKY3   GAGAGAGGGAAGATGAAGGTGAGGAGGAAGATGAGGGAACCGAGGTTTTGCTTCCAGACCAGAAGCGACGTGGATGTACT  640
Consensus          gagagagggaagatgaaggtgaggaggaagatgagggaaccgaggttttgcttccagaccagaagcgacgtggatgtact

ROC22-ScWRKY3      GGATGATGGCTACAAGTGGAGGAAGTACGGGCAGAAGGTTGTCAAGAACAGCCTCCATCCAAGGAGCTATTTCCGGTGCA  720
YC05-179-ScWRKY3   GGATGATGGCTACAAGTGGAGGAAGTACGGGCAGAAGGTTGTCAAGAACAGCCTCCATCCAAGGAGCTATTTCCGGTGCA  720
Consensus          ggatgatggctacaagtggaggaagtacgggcagaaggttgtcaagaacagcctccatccaaggagctatttccggtgca

ROC22-ScWRKY3      CTCACAGCAACTGCCGCGTGAAGAAACGGGTGGAGCGGCTGTCGACGGACTGCCGCATGGTGATGACCACGTACGAGGGC  800
YC05-179-ScWRKY3   CTCACAGCAACTGCCGCGTGAAGAAACGGGTGGAGCGGCTGTCGACGGACTGCCGCATGGTGATGACCACGTACGAGGGC  800
Consensus          ctcacagcaactgccgcgtgaagaaacgggtggagcggctgtcgacggactgccgcatggtgatgaccacgtacgagggc

ROC22-ScWRKY3      CGCCACACGCACTCTCCCTGCAGCGACGACGCTTCCTCCGCCGACCACACCGATTGCTTCAGCTCCTTCTGAATCTATCT  880
YC05-179-ScWRKY3   CGCCACACGCACTCTCCCTGCAGCGACGACGCTTCCTCCGCCGACCACACCGATTGCTTCAGCTCCTTCTGAATCTATCT  880
Consensus          cgccacacgcactctccctgcagcgacgacgcttcctccgccgaccacaccgattgcttcagctccttctgaatctatct

ROC22-ScWRKY3      ATCCCACGACCGCATACATTGACGACGCAG                                                    910
YC05-179-ScWRKY3   ATCCCACGACCGCATACATTGACGACGCAG                                                    910
Consensus          atcccacgaccgcatacattgacgacgcag
```

Figure S1. Nucleic acid sequences alignment of *ScWRKY3* in ROC22 and Yacheng05-179**.** The black color indicates the homology level of conservation of the nucleic acid sequences in the alignment at 100%.

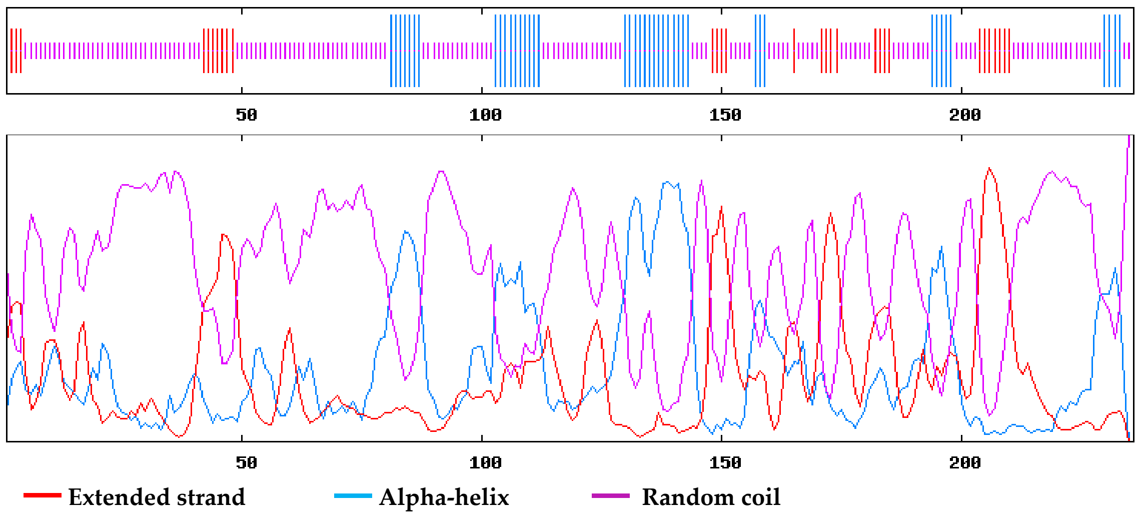

Figure S2. Secondary structure prediction of ScWRKY3. NPS@ server (https://npsa-prabi.ibcp.fr/cgi-bin/npsa_automat.pl?page=/NPSA/npsa_hnn.html) is used to predict this structure.

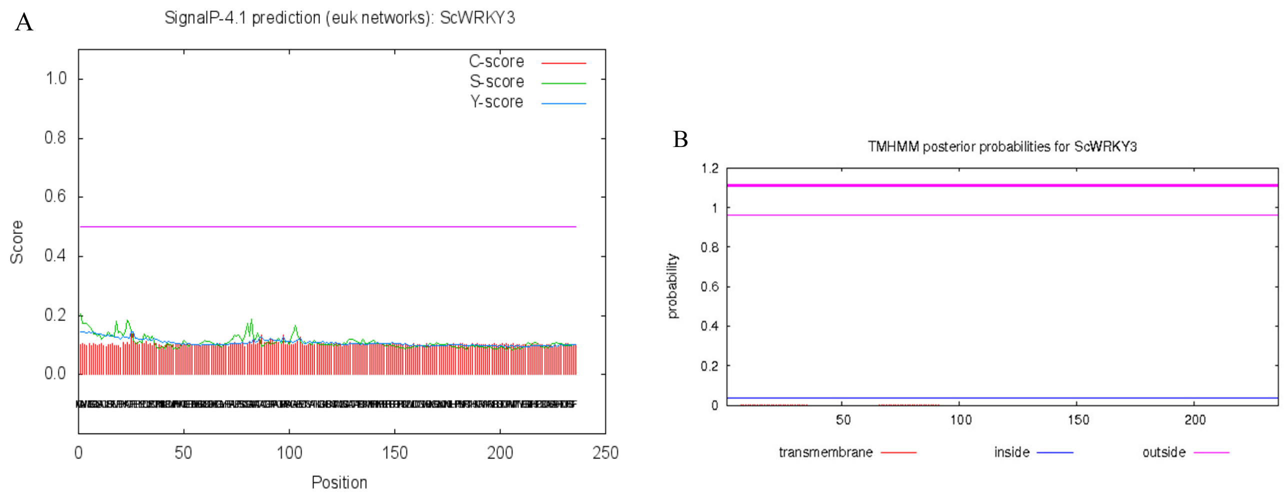

Figure S3. Signal peptide and transmembrane domain prediction of ScWRKY3. (A) SignalP 4.1 Server (http://www.cbs.dtu.dk/services/SignalP/) is used to predict the signal peptide of ScWRKY3. (B) TMHMM Server v. 2.0 (http://www.cbs.dtu.dk/services/TMHMM/) is used to predict the transmembrane domain of ScWRKY3.

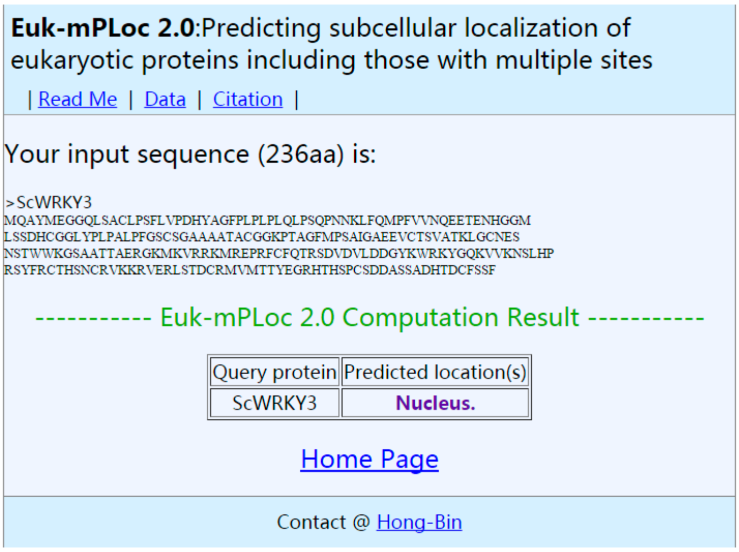

Figure S4. Subcellular localization prediction of ScWRKY3. Euk-mPLoc 2.0 Server (http://www.csbio.sjtu.edu.cn/bioinf/euk-multi-2/) is used to predict the subcellular localization of ScWRKY3.

```
A ScWRKY3   MQAYMEGGQLSACLPSFLVPDHYAGFPLPLPLQLPSQPNNKLFQMPFVVNQE.ETENHGG..MLSSDHCGGLYPLPALPFGSCSGAA.AA    86
  SbWRKY57  MQAYMEGGQLSACLPGFLVPDHYA.FPLPLPLQLPSSQ.NKLFQMPFVVDQEAETENHGGGGMLSSDHCG.LYPLPALPFGSCSGAAGAA    87
  Consensus mqaymeggqlsaclpgflvpdhyagfplplplqlpsqpnnklfqmpfvvdqeaetenhggggmlssdhcgglyplpalpfgscsgaagaa

  ScWRKY3   TACGGKPTAGFMPSAIGAEEVCTSVATKLGCNESNSTWWKGSAATT.AERGKMKVRRKMREPRFCFQTRSDVDVLDDGYKWRKYGQKVVK   175
  SbWRKY57  TACGGKPTAGFMPSAIVAEEVCTSVTTKLGCNDSNGTWWKGSAATTIAERGKMKVRRKMREPRFCFQTRSDVDVLDDGYKWRKYGQKVVK   177
  Consensus tacggkptagfmpsaigaeevctsvatklgcndsngtwwkgsaattiaergkmkvrrkmreprfcfqtrsdvdvlddgykwrkygqkvvk

  ScWRKY3   NSLHPRSYFRCTHSNCRVKKRVERLSTDCRMVMTTYEGRHTHSPCSDDASSADHTDCFSSF   236
  SbWRKY57  NSLHPRSYFRCTHSNCRVKKRVERLSTDCRMVMTTYEGRHTHSPCSDDASSADHTDCFTSF   238
  Consensus nslhprsyfrcthsncrvkkrverlstdcrmvmttyegrhthspcsddassadhtdcfssf

B ScWRKY3   MQAYMEGGQLSACLPSFLVPDHYAGFPLPLPLQLPSQPNNKLFQMPFVVNQEETENHGGMLSSDHCGGLYPLPALPFGSCSGAAAATACG    90
  MlWRKY12  ....MEGGQLSACLPGFFVPDHYAAFPLPLPLQLPSQPNNKLFQMPFVVDQEETENHGGMLSSDHCGGLYPLPALPFVSSSGAATATACG    86
  Consensus mqaymeggqlsaclpgffvpdhyaafplplplqlpsqpnnklfqmpfvvdqeetenhggmlssdhcgglyplpalpfgscsgaaaatacg

  ScWRKY3   GKPTAGFMPSAIGAEEVCTSVATKLGCNESNSTWWKGSAAATT.AERGKMKVRRKMREPRFCFQTRSDVDVLDDGYKWRKYGQKVVKNSLH   179
  MlWRKY12  GKPTAGFMPSTIGAEEVCTSVTTKLGCNESNSTWWKGSAAATIAERGKMKVRRKMREPRFCFQTRSDVDVLDDGYKWRKYGQKVVKNSLH   176
  Consensus gkptagfmpsaigaeevctsvatklgcnesnstwwkgsaaatiaergkmkvrrkmreprfcfqtrsdvdvlddgykwrkygqkvvknslh

  ScWRKY3   PRSYFRCTHSNCRVKKRVERLSTDCRMVMTTYEGRHTHSPCSDDASSADHTDCFSSF   236
  MlWRKY12  PRSYFRCTHSNCRVKKRVERLSTDCRMVMTTYEGRHTHSPCSDDASSGDHTDCFSSF   233
  Consensus prsyfrcthsncrvkkrverlstdcrmvmttyegrhthspcsddassadhtdcfssf

C ScWRKY3   MQAYMEGGQLS.A.CLPSFLVPDHYAGFPLPLPLQLPSQPNNK.LFQMPFVVNQEETENHGGMLSSDHCGGLYPLPALPFGSCSGAAAAT    87
  ZmWRKY51  MQAYMEGGQLLGACCLPGFLVPDHYAAFPLPLPLQLPSQPNDNRLFQMPFD..QEEAENHGGMLYSDQC.GLYPLPA..FGSCSAAAGAT    85
  Consensus mqaymeggqllgacclpgflvpdhyaafplplplqlpsqpndkrlfqmpfdvnqeeaenhggmlssdhcgglyplpalpfgscsaaaaat

  ScWRKY3   ACGGKPTAGFMPSAIGAEE.VCTSVATKLGCNESNSTWWKGSAATTAERGKMKVRRKMREPRFCFQTRSDVDVLDDGYKWRKYGQKVVKN   176
  ZmWRKY51  A...KPTAGFMPSTIGAETKVCTSLTTK.GCNESNSTWWKGSAATIAERGKMKVRRKMREPRFCFQTRSDVDVLDDGYKWRKYGQKVVKN   171
  Consensus acggkptagfmpsaigaeekvctslatklgcnesnstwwkgsaatiaergkmkvrrkmreprfcfqtrsdvdvlddgykwrkygqkvvkn

  ScWRKY3   SLHPRSYFRCTHSNCRVKKRVERLSTDCRMVMTTYEGRHTHSPCSDDASSADHTDCFSSF   236
  ZmWRKY51  SLHPRSYFRCTHSNCRVKKRVERLSTDCRMVMTTYEGRHTHSPCSDDASSGDHTDCFSSF   231
  Consensus slhprsyfrcthsncrvkkrverlstdcrmvmttyegrhthspcsddassadhtdcfssf

D ScWRKY3   MQAYMEGGQLSACLPSFLVPDHYAGFPLPLPLQLPSQPNNKLFQMPFVVNQEETENHGGMLSSDHCGGLYPLPALPFGSCSGAAAATACG    90
  SiWRKY12  MQAYMEGGQLGACLPSFLVPDHYA.FPLPLPLQLPSQP.NKLLQMPFD..QEESENHGVMLSSDHC.GLYQLPALPLG.CSGAASAVACG    84
  Consensus mqaymeggqlgaclpsflvpdhyagfplplplqlpsqpnnklfqmpfdvnqeesenhggmlssdhcgglyplpalpfgscsgaaaatacg

  ScWRKY3   GKPTAGFMPSAIGAEEVCTSVATKLGCNESNSTWWKGSAATTAERGKMKVRRKMREPRFCFQTRSDVDVLDDGYKWRKYGQKVVKNSLHP   180
  SiWRKY12  GKPTADFMPSTVGADEVCTSLTK..GCNDSASTWWKGSAPTMADRGKMKVRRKMREPRFCFQTRSDVDVLDDGYKWRKYGQKVVKNSLHP   172
  Consensus gkptadfmpsaigadevctslakklgcndsastwwkgsaatmadrgkmkvrrkmreprfcfqtrsdvdvlddgykwrkygqkvvknslhp

  ScWRKY3   RSYFRCTHSNCRVKKRVERLSTDCRMVMTTYEGRHTHSPCSDDASSADHTDCFSSF   236
  SiWRKY12  RSYFRCTHSNCRVKKRVERLSTDCRMVMTTYEGRHTHSPCSDDAASGDHTDCFSSF   228
  Consensus rsyfrcthsncrvkkrverlstdcrmvmttyegrhthspcsddaasadhtdcfssf

E ScWRKY3   .MQAYMEGGQLSACLPSFLVPDHYAGFPLPLPLQLPSQPNNKLFQMPFVVNQEETENHGGMLSSDHCGGLYPLPALPFGSCSGAAAATAC    89
  OsWRKY12  MHTCMEGGQLGTCLPNFYLLPDHHCMPLPPPLQLPCHP..KLLQMPFDQEDQPGI.HGVMLSSDHC.GLYPLPALPLSNSAAAAAATVA    86
  Consensus mhqameeggqlgaclpnflllddhagfplplplqlpchpnnklfqmpfdqedeegenhggmlssdhcgglyplpalpfgncaaaaaataa

  ScWRKY3   GGKPT..AGFMPSAIGAEEVCTSVATKLGCNE.....SNSTWWKGSAATTAERGKMKVRRKMREPRFCFQTRSDVDVLDDGYKWRKYGQK   172
  OsWRKY12  LGKHSAAAGSMPNIGGAEEVATTV.TKAGNESTTCNGSTTWWRGSTMAAMGEKGKMKIRRKMREPRFCFQTRSEVDVLDDGYKWRKYGQK   175
  Consensus ggkhsaaagfmpnaggaeevatsvatkagceettcngsnstwrggsaaamaekgkmkirrkmreprfcfqtrsdvdvlddgykwrkygqk

  ScWRKY3   VVKNSLHPRSYFRCTHSNCRVKKRVERLSTDCRMVMTTYEGRHTHSPCSDDASSADHTDCFSS   235
  OsWRKY12  VVKNSLHPRSYFRCTHSNCRVKKRVERLSTDCRMVITTYEGRHTHSPC.DDNSSGEHTNCFSS   237
  Consensus vvknslhprsyfrcthsncrvkkrverlstdcrmvittyegrhthspcsddassadhtdcfss

F ScWRKY3   MQAYMEGGQLSACLPSFLVPDHYAGFPLPLPLQLPSQPNNKLFQMPFVVNQEETENHGGMLSSDHCGGLYPLPA.LPFGSCSGAAAATAC    89
  ScWRKY4   ..MEGSSQLLETCLPASLYALSPCAPPHP.PLLAPLPNQHKLLQMPL.VQEPAANNHGVMLFSDHQHGGPLYPLLLPGIPFCPSAAPAT    86
  Consensus mqaegeggleaclpaflvadhpaafphplpllaplpnnklfqmpfvvneeaaenhggmlfsdhcggggplpalllfgicfcaaaapac

  ScWRKY3   GGKPTAGFMPSAIGAEEVCTSVATKLG.........CNESNSTWWKGSAATTAERGKMKVRRKMREPRFCFQTRSDVDVLDDGYKWRKYG   170
  ScWRKY4   CEKTTTGFTALDAGEAGRTSAAKAAAEIASTTTTTCNGPSSCNWWKGPAAAGEKGRMKVRRKMREPRFCFQTRSDVDVLDDGYKWRKYG   176
  Consensus cekptagffpaadaaeagctsaakaaaeiastttttcnepnscnwkggaaaaaekgkmkvrrkmreprfcfqtrsdvdvlddgykwrkyg

  ScWRKY3   QKVVKNSLHPRSYFRCTHSNCRVKKRVERLSTDCRMVMTTYEGRHTHSPCSDDASSADHTDCFSS   235
  ScWRKY4   QKVVKNSLHPRSYYRCTHSNCRVKKRVERLSEDCRMVITTYEGRHTHSPCSDDADAAGDHTGS.   240
  Consensus qkvvknslhprsyfrcthsncrvkkrverlsedcrmvittyegrhthspcsddadaaagddcfss

G ScWRKY3   ............MQAYMEGGQLSACLPSFLVPDHYAGFPLPLPLQLPSQPNNKLFQMPFVVNQEETENHGGMLSSDHCGGLYPLPALPFG    78
  Sc-WRKY   MAASLGLAHEACYAAYPPAAAASSYFPSPPPPPGDLVAEFPPAAATAMVDDYYFQFREEMGGARAPGCGGYCSPPAPVFDNGMNLLSYG    90
  Consensus maaslglaheacmaaymeaaaasacfpsflppdgdagaefplpaaapampddklfqfpeemggaeaegcggmcspdacgfdnglnalpfg

  ScWRKY3   SCSGAAAATACGGKPTA..GFMPSAIGAEEVCTSVATKLGCNESNSTWWKGSAATTAERGKMKVRRKMREPRFCFQTRSDVDVLDDGYKW   166
  Sc-WRKY   SADGDGRRLMSGPAGGNGGCRPASLIG..........................................FRTRSEVDVLDDGFKW   133
  Consensus sadgaaaalacggaggagggfmasaigaeevctsvatklgcnesnstwwkgsaattaergkmkvrrkmreprfcfqtrsdvdvlddgfkw

  ScWRKY3   RKYGQKVVKNSLHPRSYFRCTHSNCRVKKRVERLSTDCRMVMTTYEGRHTHSPCSDDASS.....ADHTDCFSSF...............   236
  Sc-WRKY   RKYGKKAVKSSPNPRNYYRCSAEGCGVKKRVERDRDDPRYVITTYDGVHNHAAPGAAYVCPPPRGASTTPCFSPPYSASSAPLVAAPSWN   223
  Consensus rkygkkavknslhprnyfrcsaegcgvkkrverdrddcrmvittydgrhnhaacgaaascppprgadhtdcfspfysassaplvaapswn

  ScWRKY3   ....................   236
  Sc-WRKY   AAFDAWKAQLHAAAAHSSESS   244
  Consensus aafdawkaqlhaaaahssess
```

Figure S5. Amino acid sequences alignment of ScWRKY3 and other WRKYs. A to G are showing the amino acid sequence alignment of ScWRKY3 and WRKY from *Sorghum bicolor* SbWRKY57 (XP_002452824.2), *Miscanthus lutarioriparius* MlWRKY12 (AGQ46321.1), *Zea mays ZmWRKY51* (XP_020393361.1), *Setaria italica* SiWRKY12 (XP_004953301.1), *Oryza sativa* OsWRKY12 (XP_015624962.1), *Saccharum* ScWRKY4 (AUV50355.1) and Sc-WRKY (ACT53875.1), respectively. All these accession numbers in brackets are from GenBank. The black and blue colors indicate the homology level of conservation of the amino acid residues in the alignment at 100 and ≥50, respectively.

Motif 1
E-value 2.8e-568
Sites 30
Width 29

Motif 2
E-value 7.6e-411
Sites 28
Width 30

Motif 3
E-value 4.9e-058
Sites 4
Width 39

Motif 4
E-value 2.5e-035
Sites 8
Width 24

Motif 5
E-value 1.3e-028
Sites 4
Width 28

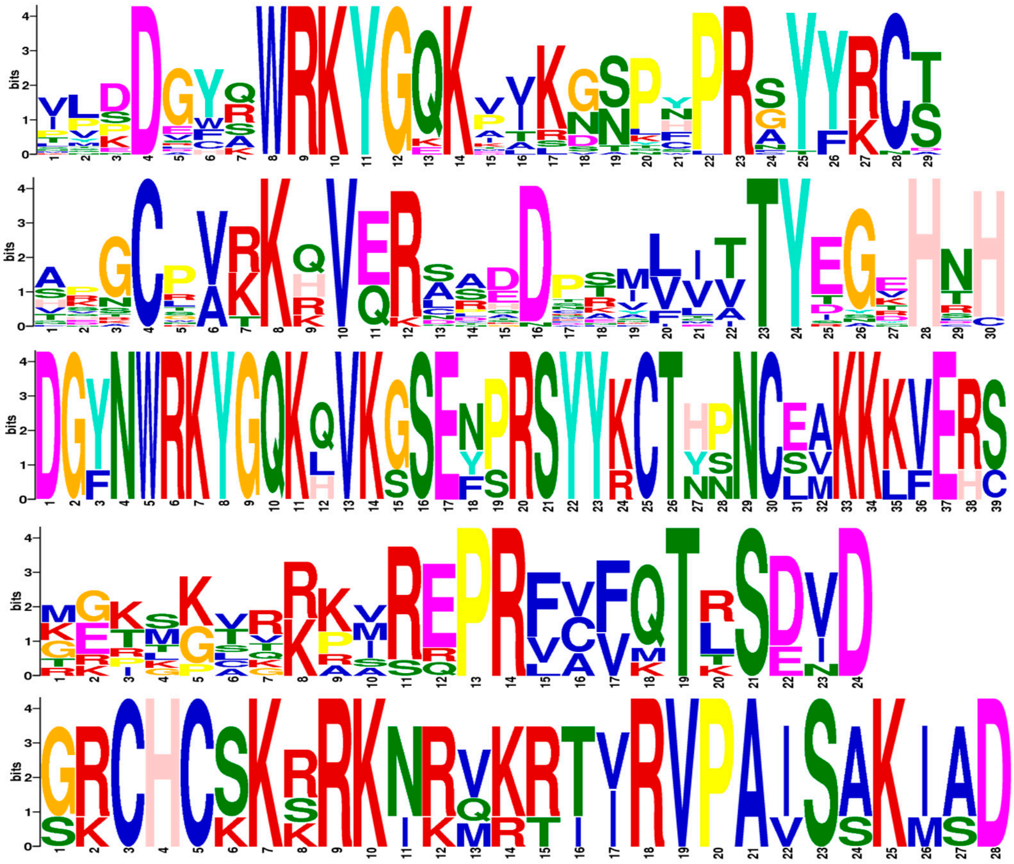

Figure S6. The logo of predicted conserved motifs in the WRKYs. The logos were predicted by the online software MEME Suite 5.0.2 (http://meme.sdsc.edu/meme/intro.html). On the y axis (measured in bits), the overall height of the stack indicating the sequence conservation at that position, while the height of symbols within the stack indicates the relative frequency of each amino acid at that position.
